# Pentosan polysulfate sodium for Ross River virus-induced arthralgia: a phase 2a, randomized, double-blind, placebo-controlled study

**DOI:** 10.1186/s12891-021-04123-w

**Published:** 2021-03-12

**Authors:** Ravi Krishnan, Melanie Duiker, Penny A. Rudd, Donna Skerrett, James G. D. Pollard, Carolyn Siddel, Rifat Rifat, Jennifer H. K. Ng, Peter Georgius, Lara J. Hererro, Paul Griffin

**Affiliations:** 1 Paradigm Biopharmaceuticals Ltd., Melbourne, Victoria Australia; 2 Institute for Glycomics, Griffith University, Southport, Queensland Australia; 3 Clinical Trials Unit, Barwon Health, Geelong, Victoria Australia; 4 Springs Medical, Daylesford, Victoria Australia; 5 Rich River Health Group, Echuca, Victoria Australia; 6 Clinical Trials Unit (Griffith Health), Griffith University, Gold Coast, Australia and Gold Coast University, Gold Coast, Queensland Australia; 7 Sunshine Coast Clinical Research, Gold Coast, Queensland Australia; 8 Department of Medicine and Infectious Diseases, Mater Misericordiae Ltd., Level 3, Aubigny Place, Raymond Terrace, South Brisbane, Queensland 4101 Australia

**Keywords:** Ross River virus, Pentosan polysulfate sodium, Pain, Arthritis, Alphavirus

## Abstract

**Background:**

Alphaviruses, such as Ross River (RRV) and chikungunya virus (CHIKV), cause significant global morbidity, with outbreaks of crippling joint inflammation and pain, leaving patients incapacitated for months to years. With no available vaccine or specific therapeutic for any alphaviral disease, and a growing economic and public health burden, there is a serious need for the development of specific therapies.

**Methods:**

This study evaluated the safety and efficacy of pentosan polysulfate sodium (PPS) in subjects with RRV-induced arthralgia in a double-blind, placebo-controlled trial. Twenty subjects were randomized 2:1 to subcutaneous PPS (2 mg/kg) or placebo (sodium chloride 0.9%) twice weekly for 6 weeks. Safety evaluation included physical examination, concomitant medications, and laboratory findings. Efficacy assessments included change from baseline in joint function (hand grip strength and RAPID3) and quality of life (SF-36) at Days 15, 29, 39 and 81 after treatment initiation. Inflammatory and cartilage degradation biomarkers were exploratory endpoints.

**Results:**

PPS was well tolerated, with a similar proportion of subjects reporting at least one treatment-emergent adverse event (TEAE) in the treatment and placebo groups. Injection site reactions were the most common TEAE and occurred more frequently in the PPS group. Dominant hand grip strength and SF-36 scores improved with PPS at all time points assessed, with hand grip strength improvement of 6.99 kg (*p* = 0.0189) higher than placebo at Day 15. PPS showed significant improvements versus placebo in adjusted mean relative change from baseline for RAPID3 Pain (*p* = 0.0197) and Total (*p* = 0.0101) scores at Day 15. At the conclusion of the study overall joint symptoms, assessed by RAPID3, showed near remission in 61.5% of PPS subjects versus 14.3% of placebo subjects. Additionally, PPS treatment improved COMP, CTX-II, CCL1, CXCL12, CXCL16 and CCL17 biomarker levels versus placebo.

**Conclusions:**

Overall, the improvements in strength and joint symptoms warrant further evaluation of PPS as a specific treatment for RRV-induced and other forms of arthritis.

**Trial registration:**

This trial is registered at the Australian New Zealand Clinical Trials Registry #ACTRN12617000893303.

**Supplementary Information:**

The online version contains supplementary material available at 10.1186/s12891-021-04123-w.

## Background

Arthritogenic mosquito-borne alphaviruses, such as chikungunya virus (CHIKV), Ross River virus (RRV) and o’nyong nyong virus (ONNV), can cause severe acute musculoskeletal inflammatory illnesses that may lead to significant muscle and joint damage. These illnesses can last months to years causing considerable pain and suffering for the patient and a significant cost to society [1].

The pathogenesis of chronic alphaviral arthritis in humans is not well understood. The isolation of viral antigen in leukocytes from joint effusions and viral RNA from synovial biopsies [2] suggest that persistent infection or viral antigen may play a role. Other evidence suggests a more independent post-viral inflammatory or autoimmune response which clinically resembles rheumatoid arthritis, with various inflammatory and immunological pathways implicated in disease pathogenesis. Several studies have demonstrated that various immune mediators are also implicated, including macrophage inhibitory factor (MIF) [3], an important cytokine in the pathogenesis of rheumatoid arthritis, and interleukin-6 (IL-6) [4].

RRV, endemic to Australia, Papua New Guinea and other South Pacific islands [5], is a common alphaviral disease with approximately 5000 cases per year in Australia [6, 7]. RRV is a debilitating, chronic alphavirus that causes rash, fever, myositis, and arthralgia that can continue or recur in peripheral joints and tissues for up to 6 years [8]. Prolonged arthralgia in RRV infection is associated with elevated levels of proinflammatory cytokines such as tumor necrosis factor (TNF)-α, interferon (INF)-γ, macrophage inflammatory protein (MIP) 1α, and various ILs [9]. This has spurred interest in studying biomarkers of inflammation and cartilage damage associated with RRV infection, their correlation with disease severity, and their response to treatment [10]. In 2015, 2017 and again in 2020, case numbers of RRV have surged with epidemics causing considerable economic and social impacts [6]. New evidence suggests that RRV has the potential to spread globally outside its endemic areas [11], and that alphaviruses such as RRV are emerging as a global threat, causing epidemics in Africa, Asia, Australia, Europe, and America [12].

Despite the significant morbidity and cost of alphaviral infection to society [1], controlling these mosquito-borne diseases is a pressing global health challenge as there is currently no vaccine to prevent any alphaviral infection nor specific treatment to reduce the duration of symptoms or alter the course of the disease. Current treatments that address the symptoms of alphaviral infections, including RRV, include a multi-factorial approach including use of local analgesics, nonsteroidal anti-inflammatory drugs (NSAIDs) and/or analgesics such as paracetamol. Corticosteroid treatment can improve outcomes but is generally not recommended as the risks likely outweigh the benefits [13]. NSAIDs are associated with gastrointestinal upset, ulcers, cardiovascular problems, bleeding problems, and liver and kidney damage, all which can limit long-term use [14]. Other treatments, such as disease-modifying antirheumatic drugs (DMARDS), can have severe adverse effects and have not been proven effective in RRV clinical trials [15].

Complicating research efforts to design a targeted therapy are significant issues with a traditional antiviral approach. In order to have a positive clinical impact, antiviral strategies have to be delivered within a very specific timeframe. With mosquito-transmitted diseases, this poses a challenge as patients are often diagnosed too late for traditional antiviral medications to be effective. Therefore, a successful treatment strategy would have to target the drivers of inflammation and disease and provide a disease modifying effect rather than target the virus itself.

Pentosan polysulfate sodium (PPS), a semisynthetic macromolecular carbohydrate derivative which chemically and structurally resembles glycosaminoglycans, has shown preclinical promise as a therapy for RRV-induced arthralgia specifically and, more generally, in alphaviral disease [16]. The oral formulation of PPS is approved for the treatment of interstitial cystitis (Australia; Elmiron®) and the injectable formulation has been in use in several European countries for prevention and treatment of thromboembolic and circulatory disorders since the 1960s. A recent study of PPS in a mouse model of RRV infection with extensive joint inflammation and thinning of articular cartilage, similar to that seen in human arthritic disease, found that PPS at a human equivalent dose (HED) of 2 mg/kg significantly reduced joint inflammation and cartilage damage. This was associated with significantly increased levels of the anti-inflammatory IL-10 and reduced levels of pro-inflammatory cytokines (IFN-γ, TNF-α, IL-2), which are typically correlated with disease severity [16].

Given the urgent need for targeted therapeutics to treat alphaviral diseases, the objective of this study was to evaluate the safety and efficacy of PPS in patients with RRV-induced arthralgia.

## Methods

### Study design

**Fig. 1:**
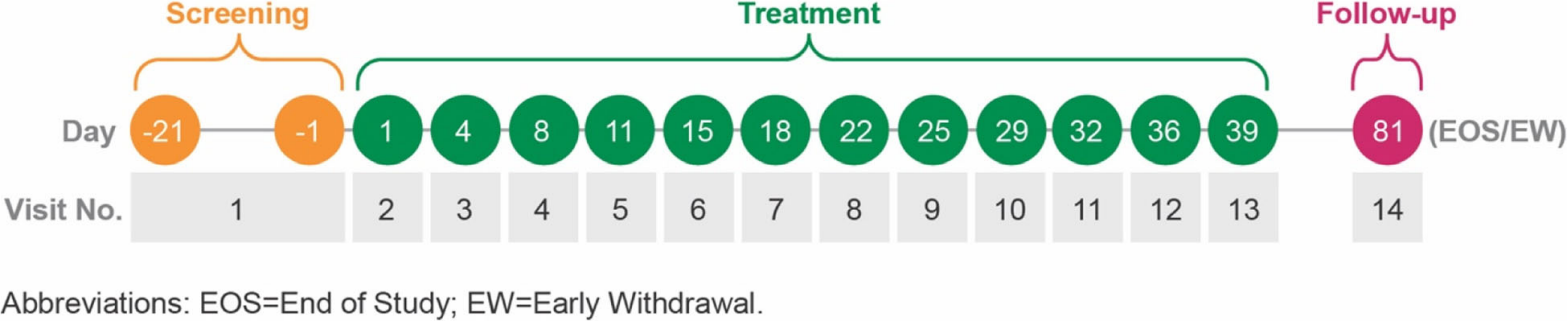
Study Design

This was a randomized, double-blind, placebo-controlled, multi-site phase 2a study (PARA-004) in patients with RRV-induced arthralgia. The study was conducted over a period of 15 months (first patient enrolled 25 Jul 2017, last visit for last patient 05 Nov 2018) at 5 clinical trial sites in Australia. The study timeline is described in Fig. 1. The study was approved by the Bellberry Human Research Ethics Committee (HREC), Adelaide, Australia and conducted in accordance with the principles of the Declaration of Helsinki, the National Health & Medical Research Council National Statement on Ethical Conduct in Research Involving Humans, and the International Council for Harmonisation of Technical Requirements for Pharmaceuticals for Human Use (ICH) Integrated Addendum To ICH E6(R1): Guideline For Good Clinical Practice E6(R2). It is registered at the Australian New Zealand Clinical Trials Registry #ACTRN12617000893303 [17].

The active drug substance, PPS, was manufactured by Bene pharmaChem and supplied by Teofarma, Valle Salimbene as a 100 mg/mL, 1 mL sterile solution. PPS was administered by slow subcutaneous injection twice weekly for 6 weeks at a dose of 2 mg/kg. The placebo consisted of sodium chloride injection BP 0.9%, manufactured by Fresenius Kabi Australia Pty Ltd. Placebo was administered by slow subcutaneous injection twice weekly for 6 weeks.

### Selection of study population

Male and female subjects aged 18 and 65 years clinically diagnosed with RRV infection according to the Australian government’s case definition [7] and with disease onset between 12 and 52 weeks prior to Day 1 were recruited. Laboratory evidence required detection by PCR and/or demonstrated seroconversions or detection of RRV IgM and RRV IgG, except if Ross River IgG was detected in a specimen collected greater than 3 months earlier. Other inclusion criteria included the involvement of at least 2 joints with swelling and tenderness, a body mass index (BMI) of 18–32 kg/m^2^ and the willingness to comply with the contraceptive requirements of the study based on the ethics guidelines by Bellberry Human Research Ethics Committee (HREC) from screening to at least 30 days after the last treatment. Exclusion criteria included documented or reported bleeding with anticoagulant or antiplatelet drugs, current treatment with any drugs that could interfere with the study including immunosuppressive or immunomodulatory drugs, evidence of any chronic condition or clinically significant illnesses having baseline coagulation parameters or platelets values outside normal ranges on Day 1 and any clinical abnormalities not related to RRV disease. Further exclusion criteria included a history of significant hypersensitivity to PPS or drugs of similar chemical or pharmacological class.

### Randomization and blinding

Subjects were randomized 2:1 to active or placebo treatment using a computer-generated random allocation sequence created by an unblinded statistician. The statistician supplied the treatment allocation list to pharmaceutical packaging professionals (PPP). Participants were enrolled by unblinded site staff, who contacted the PPP to obtain the next sequential randomization number in the study to be allocated to the participant. The randomization codes were available to the Investigator for emergency unblinding purposes. Patients, clinicians assessing outcomes, and investigators were blinded to study allocation. Study medication was drawn up by an unblinded pharmacist or nurse. PPS has a slight yellow colour and the placebo has a clear colour. Therefore, the syringe was obscured with a transparent yellow tape to mask the colour and provided in the masked syringe to the blinded clinician for injection.

## Study outcomes

### Safety assessments

The primary endpoint of the study was safety and tolerability, as assessed by adverse events (AEs), concomitant medications, clinical laboratory monitoring, physical examinations, vital signs and treatment exposure. AEs of special interest included injection site reactions and coagulation profile. Clinical laboratory testing included hematology, clinical chemistry, liver function tests, coagulation and urinalysis.

### Efficacy assessments

The secondary endpoints of the study were efficacy assessments as listed below.

Hand Grip Strength Scores: The hand grip test measured the maximum isometric strength of the hand and forearm muscles using a handgrip dynamometer. Three assessments were measured for each hand and the mean of three trials of hand grip strength for each hand recorded [18].

RAPID3 Assessment: RAPID3 is a pooled index of the 3 patient-reported American College of Rheumatology (ACR) rheumatoid arthritis (RA) Core Data Set measures: function, pain, and patient global estimate of status. Each of the 3 individual measures is evaluated for the preceding week using scores of 0 to 10, for a total of 30 [19].

Pain Scores (NRS-11): Pain was assessed at baseline and at each study visit using the Numeric Rating Scale (NRS)-11 [20], which was used to rate the intensity of pain ‘right now’ as opposed to the chronic pain response.

SF-36 Assessment: The SF-36 v2 is a 36-item, patient-reported survey of patient health, consisting of eight subscales [21]. Respondents were asked to answer the questions as they pertained to the way they felt or acted during the past week. Each scale was directly transformed into a 0–100 scale on the assumption that each question carried equal weight. A score of zero was equivalent to maximum disability and a score of 100 was equivalent to no disability.

### Biomarkers

Serum and urine samples from selected trial sites were collected at baseline and Days 15, 29, 39 and 81 post-treatment and tested for inflammatory cytokines, chemokines and biomarkers of bone and cartilage remodeling. Serum biomarkers for inflammatory cytokines and chemokines were assayed using the Bio-Plex Pro™ Human Chemokine Panel, 40-Plex kit (Bio-Rad, Gladesville, Australia) and quantified using a Bio-Plex 200® instrument (see supplementary material, Table S20, for a list of biomarkers). Quantitative analysis was performed using Bio-Plex Manager software version 6.1. Serum cartilage oligomeric matrix protein (COMP) was assayed using a sandwich ELISA (AnaMar, Lund, Sweden). Urine C-terminal telopeptides of type II collagen (CTX-II) was assayed using Urine CartiLaps® in a competitive ELISA (IDS Immuno Diagnostics System, Abacus dx Meadowbrook, Australia). Urinary CTX-II levels were normalized to total urine creatinine concentrations (R&D Systems). The mean percentage change from baseline for each biomarker was derived by dividing the maximum quantitated biomarker concentration reached during the period of the trial by its baseline concentration.

### Sample size calculation and statistical analysis

As this was a pilot study and descriptive in nature, no formal statistical sample size estimation was performed. Rather, the sample size was based on clinical and practical considerations. No formal hypothesis testing was planned to examine treatment effects. However, changes over time for the secondary efficacy outcomes were explored using mixed models for repeated measures (MMRM). All efficacy analyses were performed on the intention to treat (ITT) and per protocol (PP) populations. Changes over time in efficacy endpoints were explored by summary statistics, time trend plots and mixed effects models. Level of significance for efficacy analyses was *p* < 0.05. All confidence intervals (CI) were constructed at a 95% CI. Analysis was performed using Statistical Analysis System (SAS) Version 9.4. For biomarker analysis, statistical significance was determined using an unpaired parametric T-test with GraphPad Prism version 8.0.

## Results

### Subject disposition and demographics

Twenty (20) eligible subjects were enrolled into the study and randomized to PPS (13 subjects) or placebo (7 subjects). The age of subjects was similar between groups, with a mean (SD) of 47.6 (6.53) years overall. Overall, 9 (45.0%) subjects were male and 11 (55.0%) subjects were female. There was a slightly higher proportion of female subjects in the PPS group (8 subjects, 61.5%) compared to the placebo group (3 subjects, 42.9%). All subjects were Caucasian. Height, weight and BMI were similar between groups. See Table S1 for a summary of demographic and baseline characteristics for the ITT (all randomized subjects)/Safety population (all subjects who received at least one dose of PPS or placebo).

Two subjects (15.4%) from the PPS treatment group were excluded from the PP population due to early termination, which included one subject due to an AE (injection site erythema) and one other subject due to withdrawal of consent (because of needle phobia) resulting in 11 PPS subjects (84.6% of ITT) and 7 placebo subjects (100% of ITT) in the PP population. See Fig. S1 for a diagram of subject disposition.

The most commonly affected joints at baseline were the wrist for the PPS group (12 subjects, 92.3%), and the hand (including fingers) for the placebo group (6 subjects, 85.7%). A notably higher proportion of subjects in the PPS group (12 subjects, 92.3%) reported affected wrist joints at baseline compared to the placebo group (2 subjects, 28.6%). Similarly, a notably higher proportion of subjects in the PPS group (11 subjects, 84.6%) reported affected knee joints at baseline compared to the placebo group (2 subjects, 28.6%).

### Adverse events

Overall, 19 (95.0%) subjects across both treatment groups reported a total of 152 treatment-emergent adverse events (TEAEs), with the proportion of subjects reporting at least one TEAE being similar between groups (Table 1). The most frequently reported TEAE was injection site reactions, specifically injection site bruising and hemorrhage. The majority of injection site reactions (93.5%) were observed in the PPS group, and were mild in severity, considered definitely related to study drug and recovered/resolved. There was one TEAE (injection site erythema) reported by a subject in the PPS group which led to premature discontinuation of study drug (Table 1). There were no serious AEs (SAEs) reported during the study.

**Table 1:**
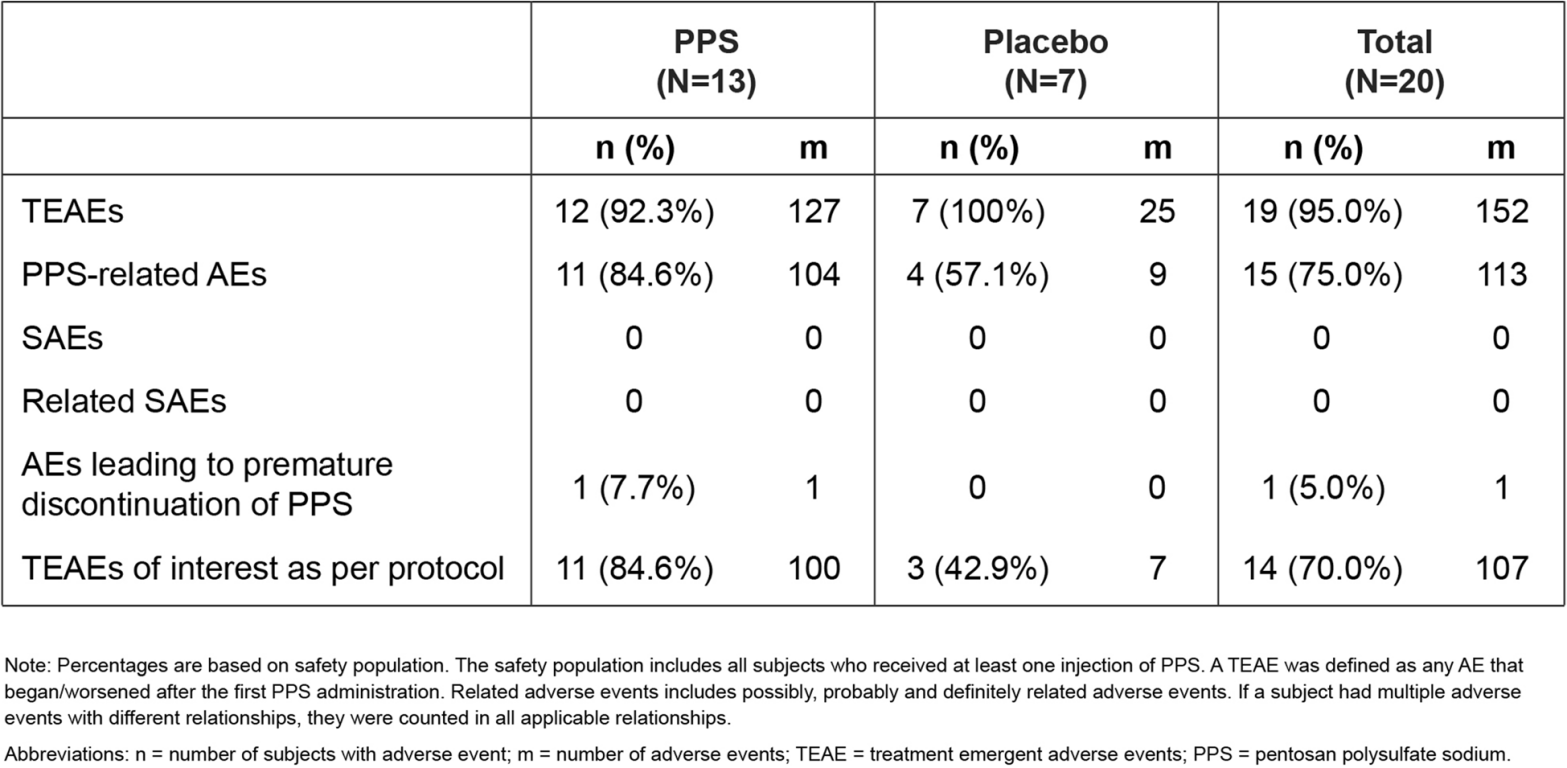
Summary of Treatment-Emergent Adverse Events by Treatment Group

The majority of laboratory values over the course of the study were similar between groups, with no identifiable changes or trends over time. Clinical chemistry values over the course of the study were similar between groups, except lipase, which increased in the PPS group between baseline and Day 39, but then returned to near baseline values at Day 81. There were no notable differences between treatment groups in liver function test (LFT) parameters except aspartate aminotransferase (AST) and alanine aminotransferase (ALT). In the PPS group, AST and ALT increased between baseline and Day 39 (the highest value was 1.4 x upper limit of normal [ULN] for AST and 2.7 x ULN for ALT), and then decreased slightly at Day 81. There were no notable changes in AST and ALT results for the placebo group. The LFT elevations observed in this study are consistent with the known safety profile of PPS.

### Hand grip strength scores

**Fig. 2:**
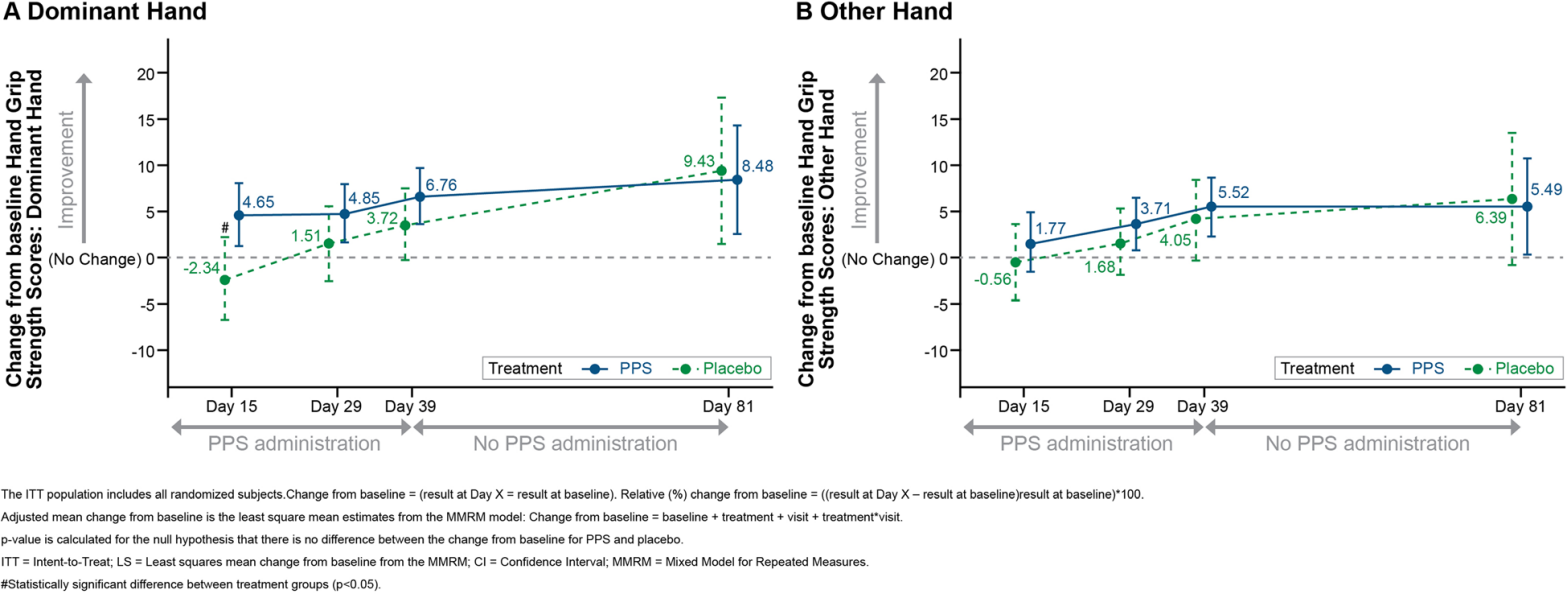
Hand Grip Strength Scores (kg), Adjusted Mean Change from Baseline: ITT Population

In the PPS group, there were increases in hand grip strength (measured in kg) between baseline and all time points for the dominant hand, and at Days 29, 39 and 81 for the other hand (Fig. 2). In the placebo group, mean hand grip strength decreased slightly between baseline and Day 15 for both hands, and then increased through to Day 81. The minimal clinically important difference (MCID) of 6.8 kg [22] was achieved by the PPS group at Days 39 and 81 for the dominant hand, and by the placebo group at Day 81 for the dominant hand. A maximum increase in mean hand grip strength of 94.48% was observed in the PPS group at Day 81 for the other hand (Table S2). Magnitude of change from baseline was consistently higher in the PPS group compared to placebo, with the difference between groups being statistically significant at Day 15 for the dominant hand. At Day 81, hand grip strength scores remained higher in both the PPS and placebo groups compared to baseline (Fig. 2).

### NRS pain scores

**Fig. 3:**
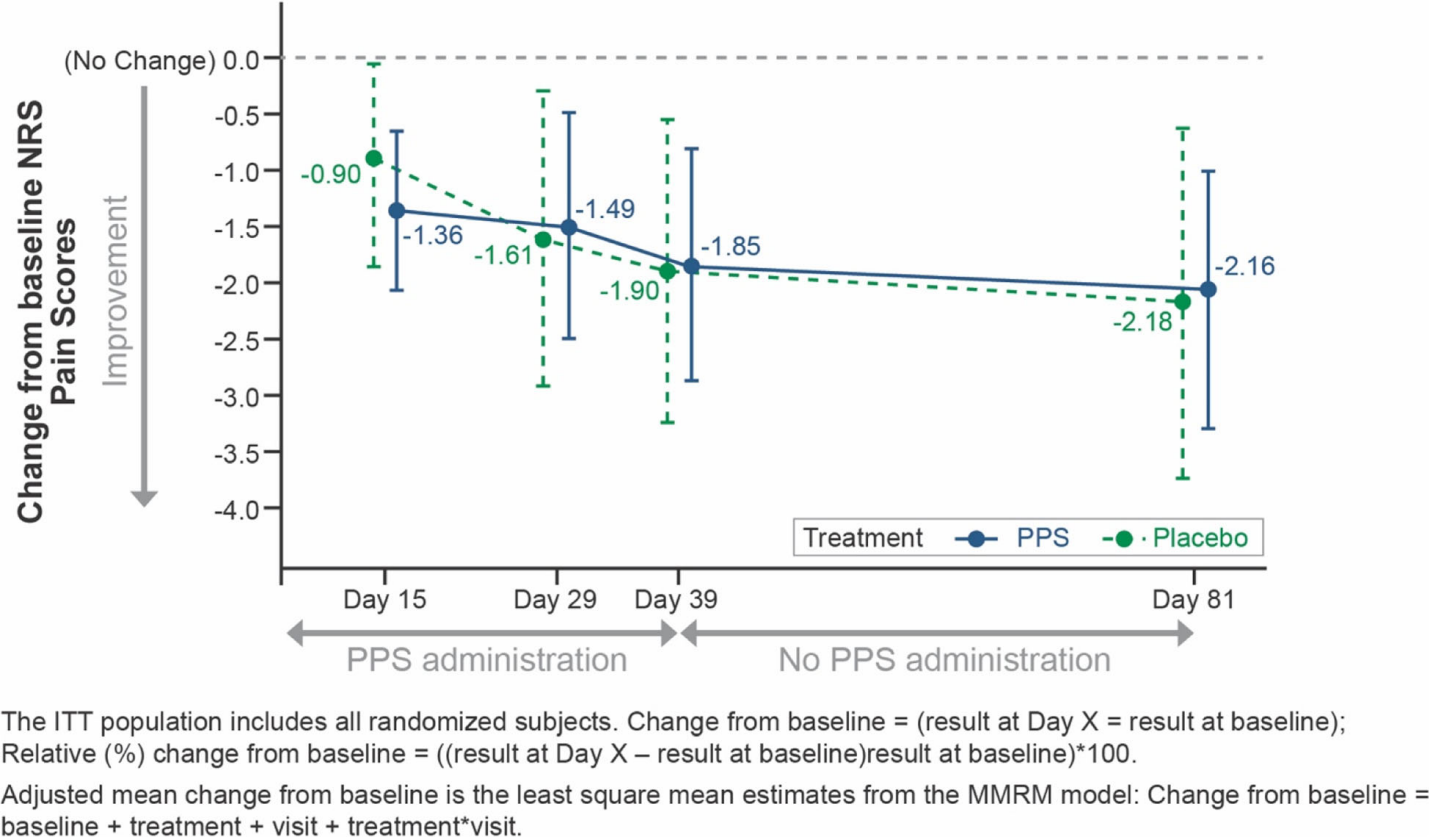
NRS Pain Scores, Adjusted Mean Relative Change from Baseline: ITT Population

In the PPS group, there were decreases in NRS Pain scores, representing pain reduction, between baseline and all time points for adjusted mean change (Fig. 3), and at Days 15 (− 30.7%) and 81 (− 50.55%) for adjusted mean relative change from baseline (Table S3). In the placebo group, there were decreases in NRS Pain scores between baseline and Days 29, 39 and 81 for adjusted mean change (Fig. 3). Differences between PPS and placebo groups were not statistically significant at any timepoint.

### RAPID3 scores

**Fig. 4:**
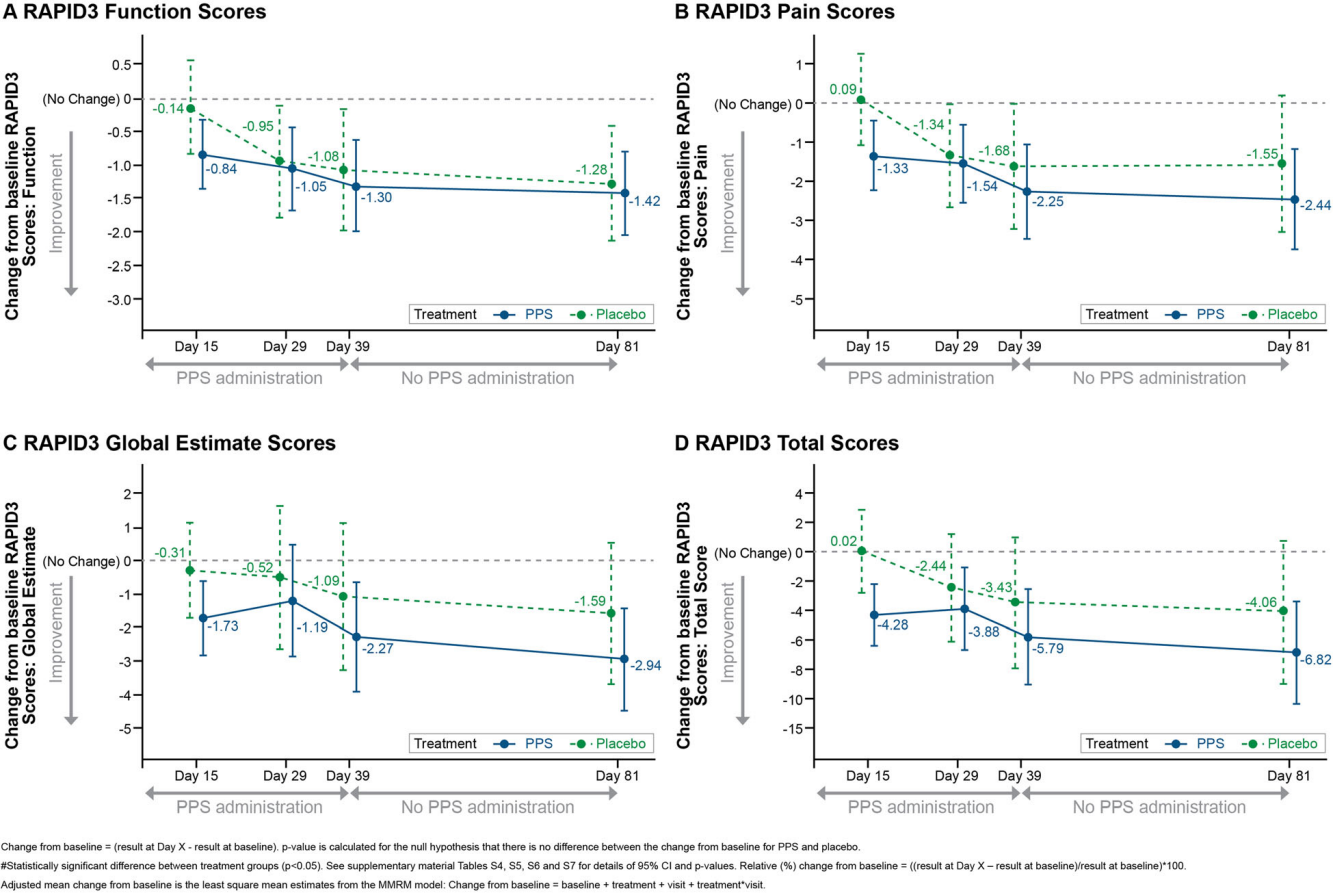
RAPID3 Scores, Adjusted Mean Change from Baseline: ITT Population

RAPID3 Function, Pain, Global Estimate and Total scores are shown in Fig. 3 and Tables S4 to S8. In the PPS group, decreases in RAPID3 Function scores were observed over time, representing improved function (Fig. 4). In the placebo group, similar decreases in Function scores were observed; however, magnitude of change from baseline was consistently higher in the PPS group compared to placebo, with no significant differences between PPS and placebo groups. A maximum decrease in mean RAPID3 Function score of 64.6% was observed in the PPS group at Day 81 (Table S4); however, the difference between the PPS and placebo groups was not significant.

RAPID3 Pain scores decreased in the PPS group over time, representing pain reduction (Fig. 4, Table S5). Smaller reductions in RAPID3 Pain scores were observed in the placebo group over time. The adjusted mean relative change from baseline was significantly higher in the PPS group (− 28.05%) versus placebo (9.9%) at Day 15 (*p* = 0.0197).

In the PPS group, there were decreases in RAPID3 Global Estimate scores over time (Fig. 4, Table S6), representing improved patient global estimate status. In the placebo group, RAPID3 Global Estimate scores demonstrated variable changes over time, and there were no statistically significant differences between treatment groups at any time point.

RAPID3 Total scores decreased significantly over time in the PPS group, representing an overall improvement in symptoms (Fig. 4, Table S7). In the placebo group, less pronounced decreases in RAPID3 Total scores were observed, with magnitude of adjusted mean change from baseline (*p* = 0.0223) and adjusted mean relative change from baseline (*p* = 0.0101) being significantly higher in the PPS group compared to the placebo group at Day 15. The MCID of 3.8 [23] was achieved by the PPS group at all time points and was not achieved at any time point by the placebo group.

### RAPID3 severity categories

At baseline, a higher proportion of subjects in the placebo group (4 subjects, 57.1%) were in the ‘moderate severity’ category compared to the PPS group (3 subjects, 23.1%), while more subjects in the PPS group (7 subjects, 53.8%) were in the ‘high severity’ category compared to placebo (2 subjects, 28.6%); see Table S8.

In the PPS group, there was a notable trend for subjects to shift from the ‘moderate severity’ and ‘high severity’ categories to the ‘low severity’ and ‘near remission’ categories over time, though some subjects in both groups did shift from lower to higher severity. At Day 81, 1 (7.7%) PPS subject was in the ‘moderate severity’ category, compared to 3 (23.1%) PPS subjects at baseline. Similarly, at Day 81 there were only 2 (15.4%) PPS subjects in the ‘high severity’ category, compared to 7 (53.8%) PPS subjects at baseline.

There were some transient shifts in severity category over time in subjects from the placebo group, however there was no identifiable trend.

### SF-36 scores

**Fig. 5:**
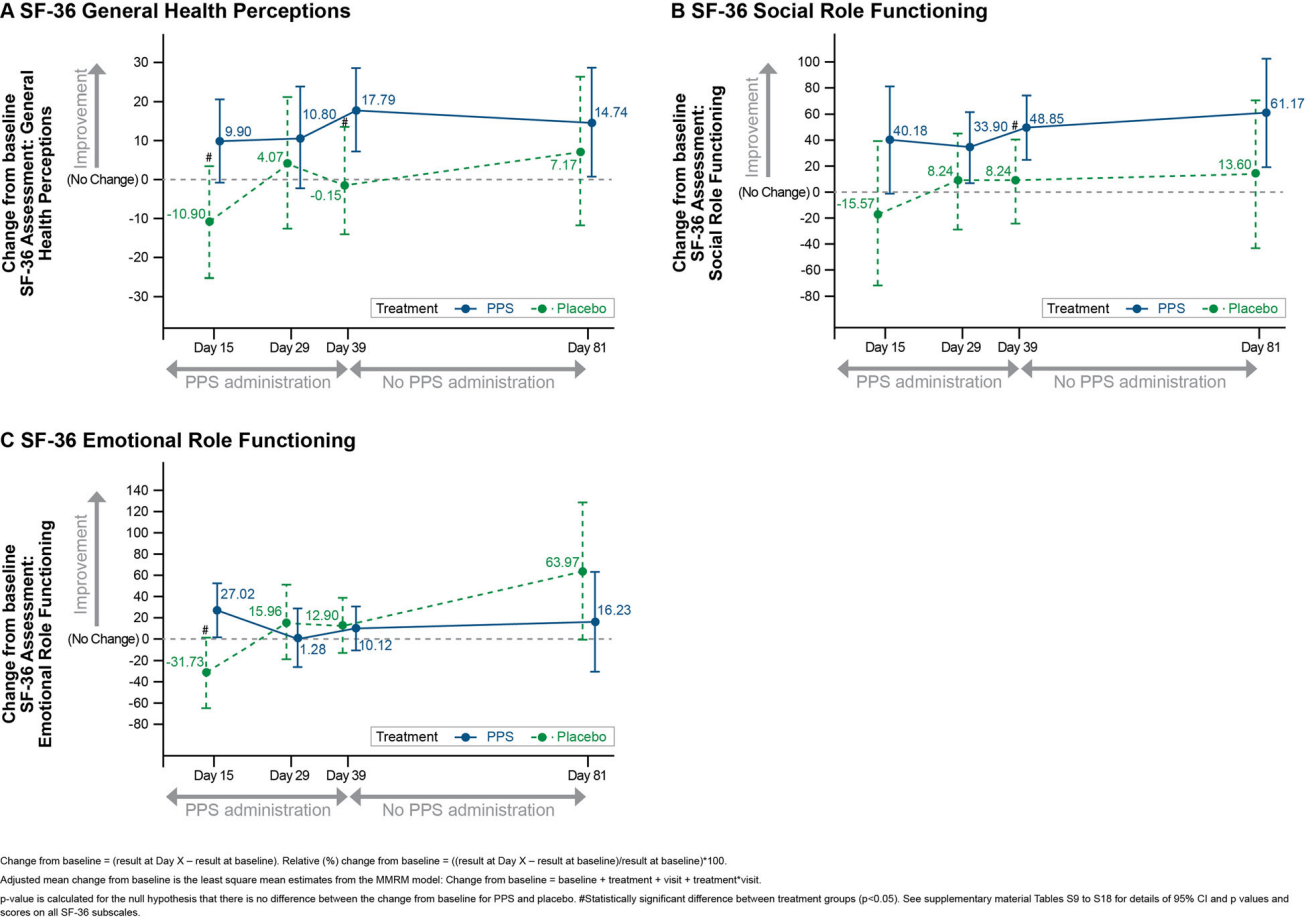
SF-36 Domain Scores, Adjusted Mean Change from Baseline: ITT Population

In the PPS group, improvements from baseline were observed on many SF-36 scores (Tables S9-S18), but for most SF-36 scores, the differences between PPS and placebo did not achieve statistical significance. However, the adjusted mean relative change from baseline for PPS versus placebo was statistically significant for General Health Perceptions, Social Role Functioning and Emotional Role Functioning in the ITT population (Fig. 5). In the PP population, adjusted mean relative change from baseline for PPS versus placebo in Bodily Pain scores was statistically significant at Days 15, 39, and 81 (Table S12).

### Biomarker analysis

Not all trial sites were able to process samples for biomarker analysis, so sample collection was restricted to 14 of 20 subjects. Of the 14 participants with biomarker samples, 13 had baseline samples which were measurable, 8 in the PPS group and 5 in the placebo group. Of 40 serum inflammatory biomarkers tested, 3 (C-C Motif Chemokine Ligand 1 [CCL1], *p* = 0.0092; C-X-C Motif Chemokine Ligand 12 (CXCL12), *p* = 0.0357; and C-C Motif Chemokine Ligand 17 [CCL17], *p* = 0.034) demonstrated a statistically significant reduction in the PPS group compared to placebo (Fig. S2). In contrast, C-X-C Motif Chemokine Ligand 16 (CXCL16) showed a statistically significant increase (*p* = 0.019) with PPS treatment compared to placebo. The bone and cartilage remodeling biomarkers serum cartilage oligomeric matrix protein (COMP; *p* = 0.0489) and urine C-terminal cross-linked telopeptide of type II collagen (CTX-II; *p* = 0.017) showed statistical significance in reduced levels with PPS compared to placebo (Fig. S2).

## Discussion

Alphaviruses such as RRV and CHIKV cause incapacitating joint disease ranging from mild arthralgia to severe, debilitating arthritis, with redness, swelling and synovial effusions. In the past few years studies have identified many similarities between the pathobiology of infectious arthritis and incapacitating rheumatoid arthritis (RA) [3, 5, 24]. Similarities include destruction of articular cartilage and ankylosis of the joints, leading to disability, decreased quality of life and other comorbidities further highlighting the need for specific treatments to curtail the course of disease.

This study demonstrated that PPS administered subcutaneously at a dose of 2 mg/kg twice weekly for 6 weeks is well tolerated and has the potential to improve pain and function in subjects with RRV-induced arthralgia. Injection site reactions were the most commonly reported PPS-related TEAE, which are a known side-effect of PPS injections. Increases in AST and ALT were anticipated based on previous studies of PPS. The safety profile of PPS in this study at this dose is consistent with the previously observed tolerance profile of PPS, with injection site reactions such as mild bruising being the most commonly reported AE [25, 26].

PPS treatment was associated with significant improvement in pain as measured by the adjusted mean relative change from baseline in RAPID-3 Pain score and RAPID-3 Total score (which measures pain and function) at Day 15. PPS also significantly improved the objective endpoints of hand grip strength, a clinically important measure of function, and biomarker analysis. PPS produced statistically significant changes versus placebo in 6 novel biomarkers, COMP, CTX-II, CCL1, CXCL12, CXCL16 and CCL17. The serum biomarkers COMP and CTX-II were reduced with PPS compared to placebo, indicating that PPS may inhibit the degenerative process in the joints [27, 28] of RRV subjects. The biomarkers CCL1 [29], CXCL12 [30] and CCL17 [31], which are chemokines involved in synovial recruitment of inflammatory cells, were reduced in PPS-treated subjects compared to placebo. In contrast, CCL16 [32], which is involved in bone integrity by recruiting osteoblasts, was increased in PPS-treated subjects, compared to placebo. This suggests the possible role PPS may play in reducing inflammation and supporting bone integrity, which could help explain the improvements seen in hand grip strength.

Some aspects of QOL, as assessed by SF-36 General Health Perceptions, Social Role Functioning, and Emotional Role Functioning scores, showed statistically significant improvements with PPS versus placebo. Additionally, despite not achieving statistical significance, improvements in functional status assessed in the RAPID3, and QOL as assessed by other SF-36 scores were reported. This may be attributed to the anti-inflammatory actions of PPS that inhibit NF-ĸβ activation of pro-inflammatory cytokines [33], and chondroprotective effects by blocking cartilage and bone degeneration via aggrecan degrading enzymes such as a disintegrin and metalloproteinase with thrombospondin motifs (ADAMTS)-4, ADAMTS-5 [34] and matrix metalloproteinases (MMP) such as MMP3 [33]. The combined anti-inflammatory actions of PPS via targeting NF-ĸβ and the inhibited expression of pain mediators of bone and cartilage cells [35] may have a direct effect on this observed early pain reduction response. In the preclinical study involving RRV-induced arthralgia in a mouse model [16], it was demonstrated that PPS improved the clinical disease scores in RRV-infected mice and reduced cartilage damage without affecting viral clearance. The anti-inflammatory and anti-arthritic actions of PPS have also been demonstrated in a study by Wijekoon et al. [36] focused on collagen-induced arthritis in rats.

The positive safety and efficacy results from this initial dose may warrant exploration of the impact of an increased dose. The subcutaneous dose of 2 mg/kg used in this study was based on the safety and tolerability of PPS observed in an open-label trial of patients with mild radiographic knee osteoarthritis who were administered 6 weekly subcutaneous injections of PPS at 2 mg/kg [26] and the 2 mg/kg twice weekly intramuscular injections reported by Sampson et al. [37] which demonstrated improved clinical outcomes of pain and function and reduction in bone marrow edema lesions. However, a weekly IM dose of 3 mg/kg was well tolerated in a randomized double-blind placebo-controlled clinical trial of patients with knee osteoarthritis in which a statistically significant difference was seen in proportion of patients in the treatment group who experienced improvement in pain on walking as compared to the control group [25]. Assessing the use of an alternate dosing regimen in the RRV patients may result in an increased impact on biomarkers and other scores that did not achieve statistical or clinical significance at 2 mg/kg.

A limitation of our study is the potential impact a placebo effect may have on subjective endpoints such as pain and function. In a meta-analysis of pain and function placebo responses in patients with osteoarthritis, Huang et al. [38] calculated a “placebo response ratio” of 0.44 (where 0 means the placebo effect made no contribution to the response and 100 means all the response is due to placebo effect). Combining this with the subjective nature of evaluating pain may have resulted in our failing to achieve statistical significance in all but one of the patient-reported outcome pain measures used. However, achieving statistical significance in the objective biomarker and hand grip strength as well as some measurements of QOL and one subjective measure of pain illustrates the impact of treatment and may provide guidance on future study design. Other limitations of the study include the small number of subjects examined due to the rarity of the disease and the lack of follow-up beyond 81 days. That all subjects were Caucasian also limits the applicability.

## Conclusions

With alphaviral arthritis causing a significant global disease burden and no licensed antivirals, targeted therapies or vaccines, compounded by the difficulties of classic antiviral strategies, the findings of this pilot study support continued evaluation of PPS as a disease modifying therapy for the improvement of RRV-induced arthralgia and other viral arthralgias.

## Supplementary Information

**Additional file 1.**

## Data Availability

All data generated or analysed during this study are included in this published article (and its supplementary information files).

## Abbreviations

ALT: Alanine aminotransferase
AST: Aspartate aminotransferase
CHIKV: Chikungunya virus
CTX-II: C-terminal telopeptides of type II collagen
DMARD: Disease-modifying antirheumatic drug
HED: Human equivalent dose
HREC: Human Research Ethics Committee
ICH: International Council for Harmonisation of Technical Requirements for Pharmaceuticals for Human Use
INF: Interferon
ITT: Intention to treat
LFT: Liver function test
MIF: Macrophage inhibitory factor
MIP: Macrophage inflammatory protein
MMP: Matrix metalloproteinases
NSAID: Nonsteroidal anti-inflammatory drug
ONNV: O’nyong nyong virus
PP: Per protocol
PPP: Pharmaceutical packaging professionals
PPS: Pentosan polysulfate sodium
RRV: Ross River virus
SAE: Serious adverse events
TEAE: Treatment-emergent adverse event
TNF: Tumor necrosis factor
ULN: Upper limit of normal
