# Supplementary material for: Pentosan polysulfate sodium for Ross River virus-induced arthralgia: a phase 2a, randomized, double-blind, placebo-controlled study
Source: BMC Musculoskelet Disord. 2021 Mar 12;22:271. doi: 10.1186/s12891-021-04123-w (PMC7955617; doi:10.1186/s12891-021-04123-w)

**Title:**  Pentosan Polysulfate Sodium for Ross River Virus-Induced Arthralgia: A Phase 2a, Randomized, Double-Blind, Placebo-Controlled Study

**Authors:** Ravi Krishnan^1^ PhD, Melanie Duiker^1^ BASc, Penny A. Rudd^2^ BSc, MSc, PhD, Donna Skerrett^1^ MD, MS, James G.D. Pollard^3^ MBBS, FRACP, Carolyn Siddel^4^ BSc, BSc (Hons), MBBS, Rifat Rifat^5^ MD, FACRRM, FAAFP, Jennifer H.K. Ng^6^ MD, MB BS, MRCP, FRACP, Peter Georgius^7^ MBBS, BMedSc, Lara J. Hererro^2^† BSc (Hons), Grad Dip Ed, MD, PhD and Paul Griffin^8^*† BSc (Hons), MBBS, FRACP, FRCPA

**Affiliations:**

1. Paradigm Biopharmaceuticals Ltd., Melbourne, Australia.
2. Institute for Glycomics, Griffith University, Southport, Qld, Australia.
3. Clinical Trials Unit, Barwon Health, Geelong, Victoria, Australia.
4. Springs Medical, Daylesford, Australia.
5. Rich River Health Group, Echuca, Australia.
6. Clinical Trials Unit (Griffith Health), Griffith University, Gold Coast, Australia and Gold Coast University, Australia.
7. Sunshine Coast Clinical Research, QLD, Australia.
8. Mater Misericordiae Ltd., QLD, Australia.

*Corresponding author:

Paul Griffin

Department of Medicine and Infectious Diseases, Mater Misericordiae Ltd.

Level 3, Aubigny Place, Raymond Terrace, South Brisbane QLD 4101, Australia

[paul.griffin@mater.org.au](https://exchange.robsoninc.com/owa/redir.aspx?C=BJCTYQOdBNq5heufgfFEFCGAQPy7pHJRPVw_2LWoUX2mmOmBlBvYCA..&URL=mailto%3apaul.griffin%40mater.org.au)

† Contributed equally to this work and should be considered joint senior author

Supplementary Materials

*Supplementary figures*


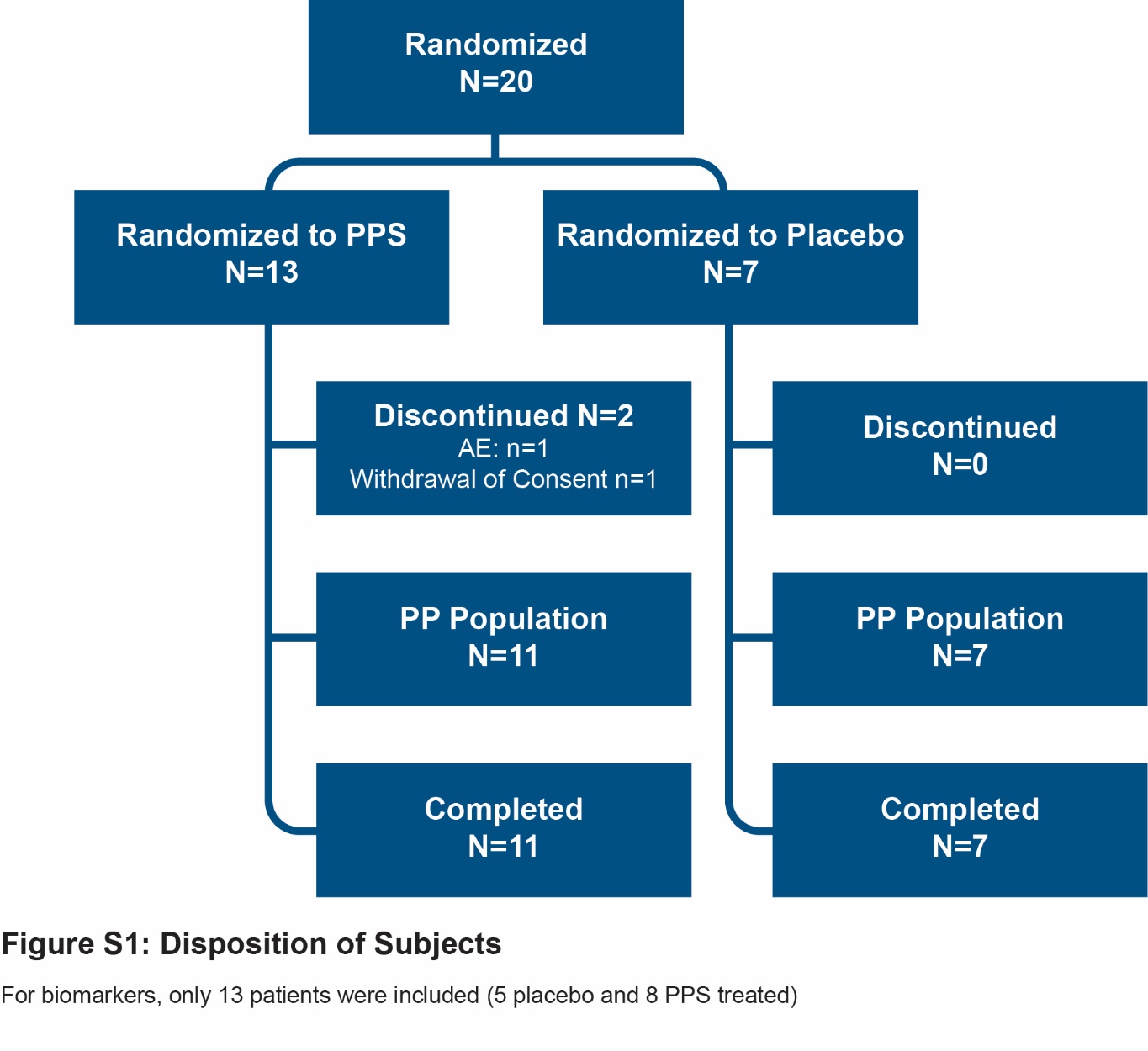


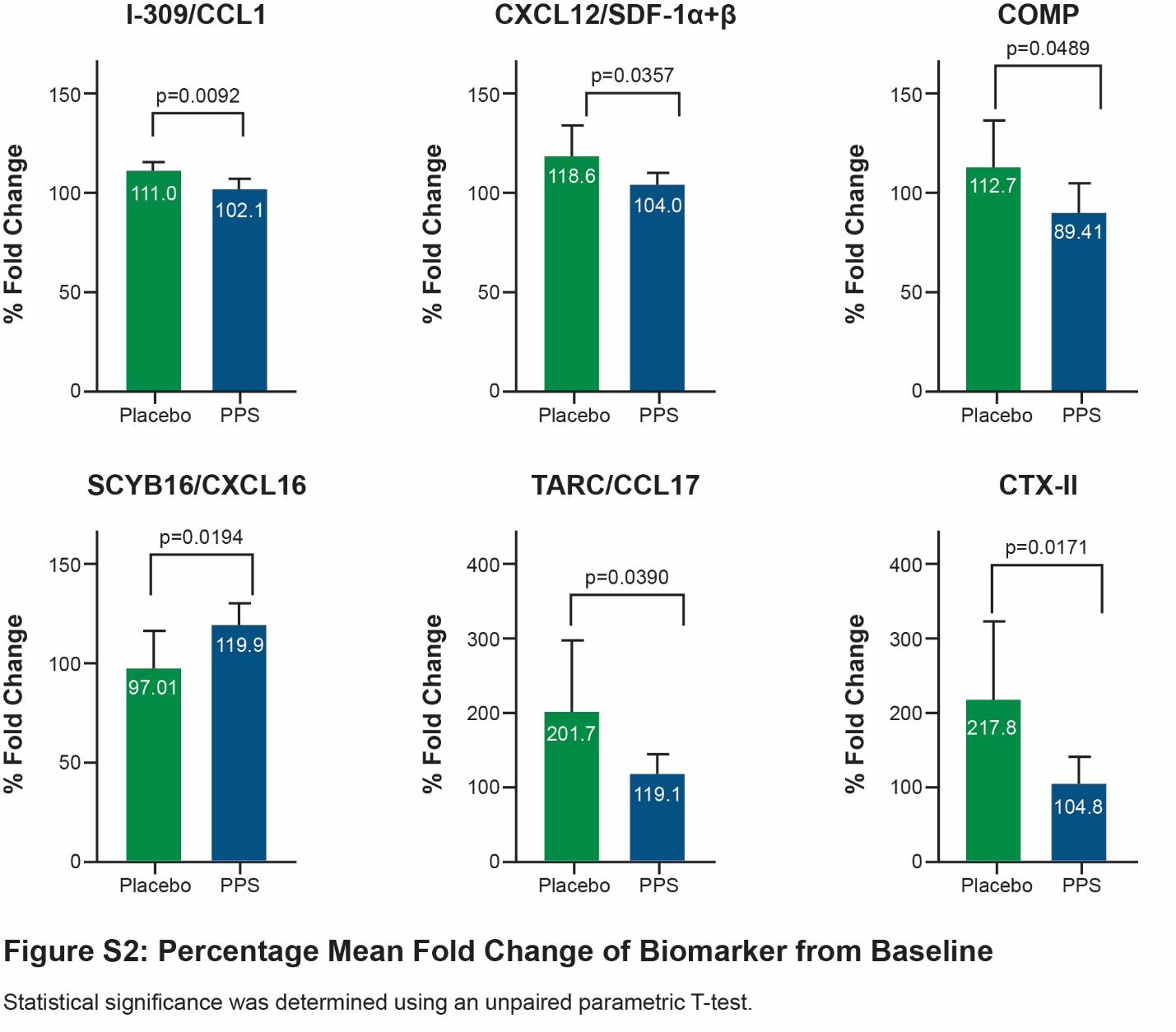


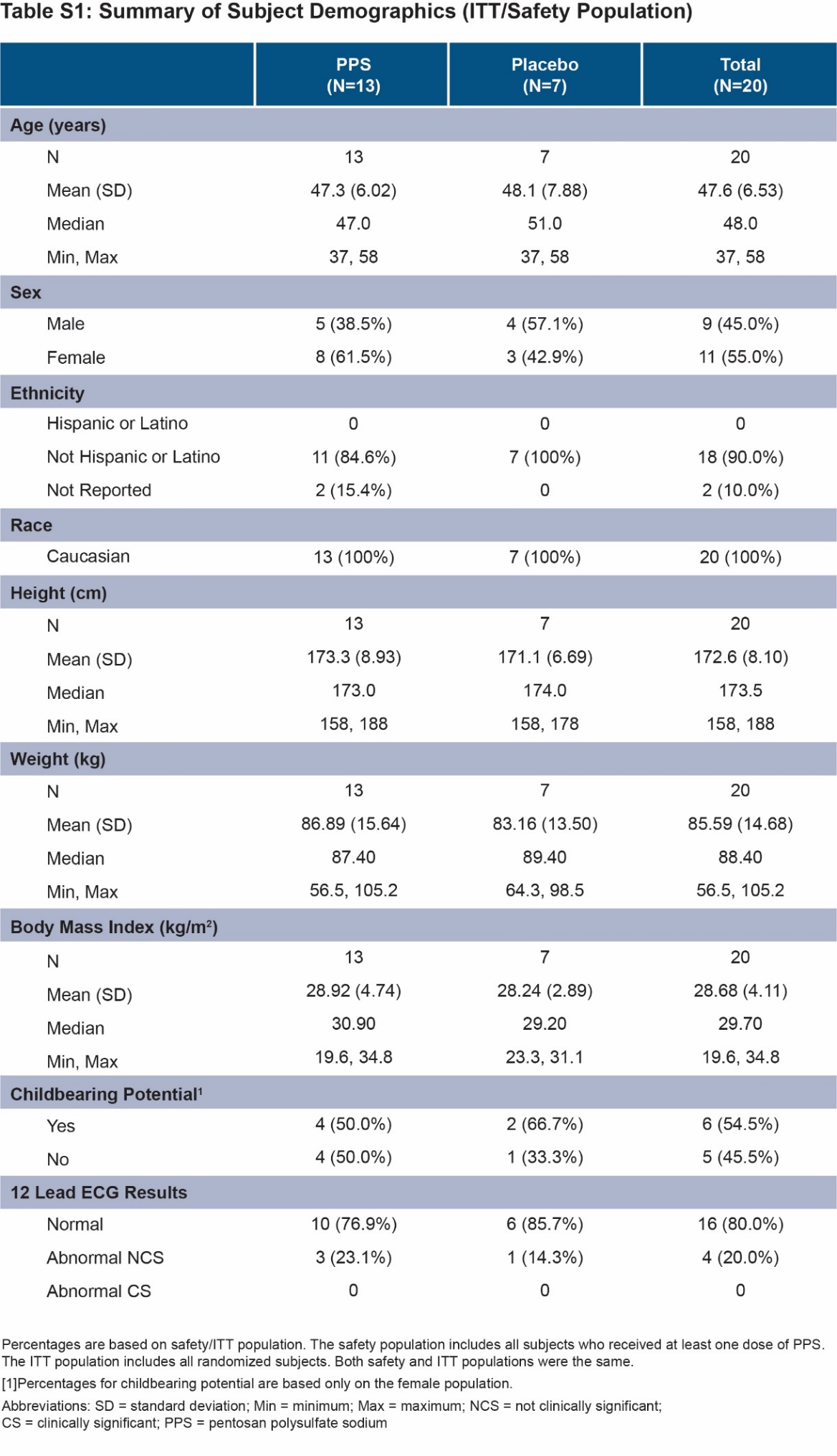


*Supplementary tables*


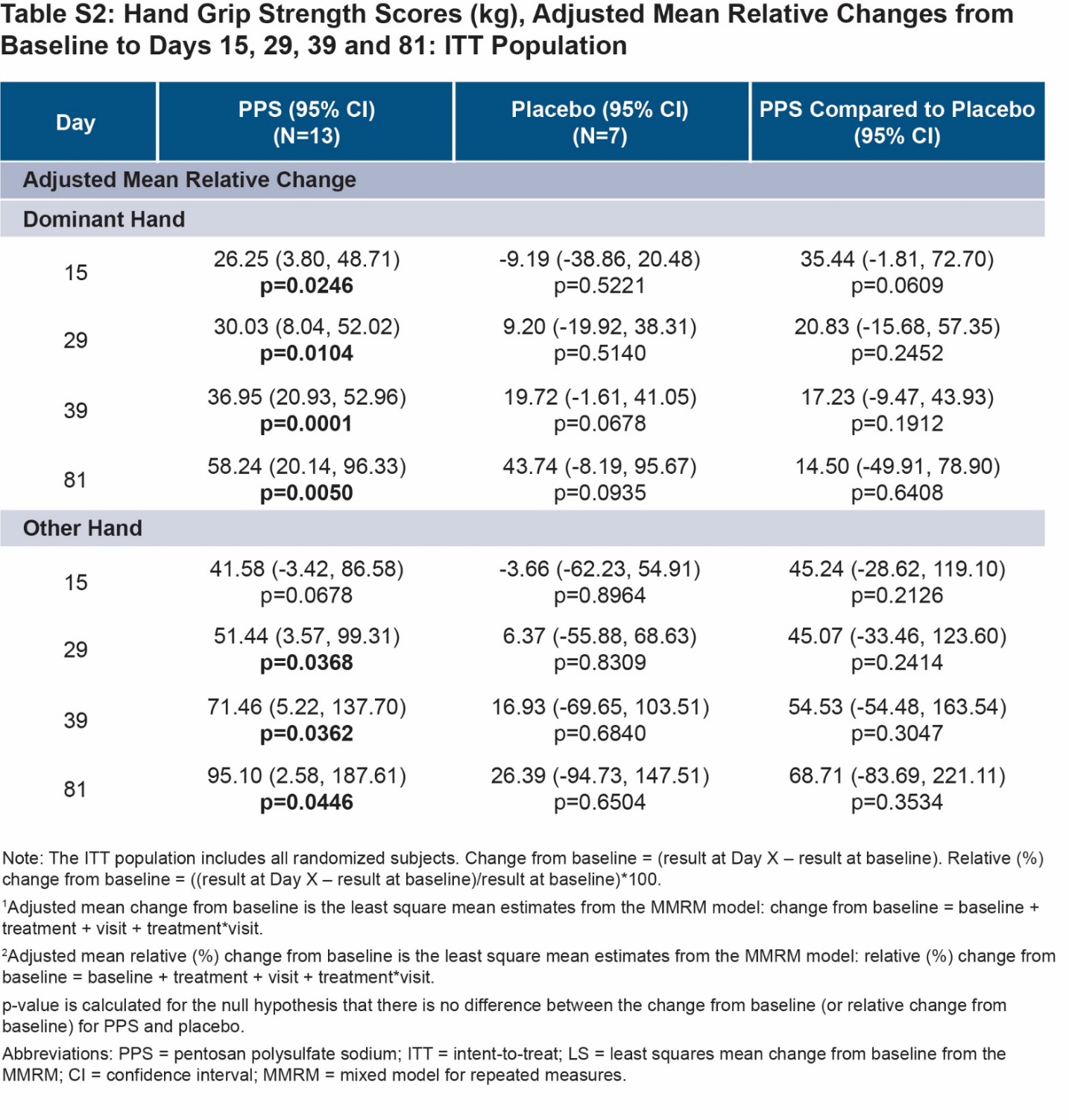


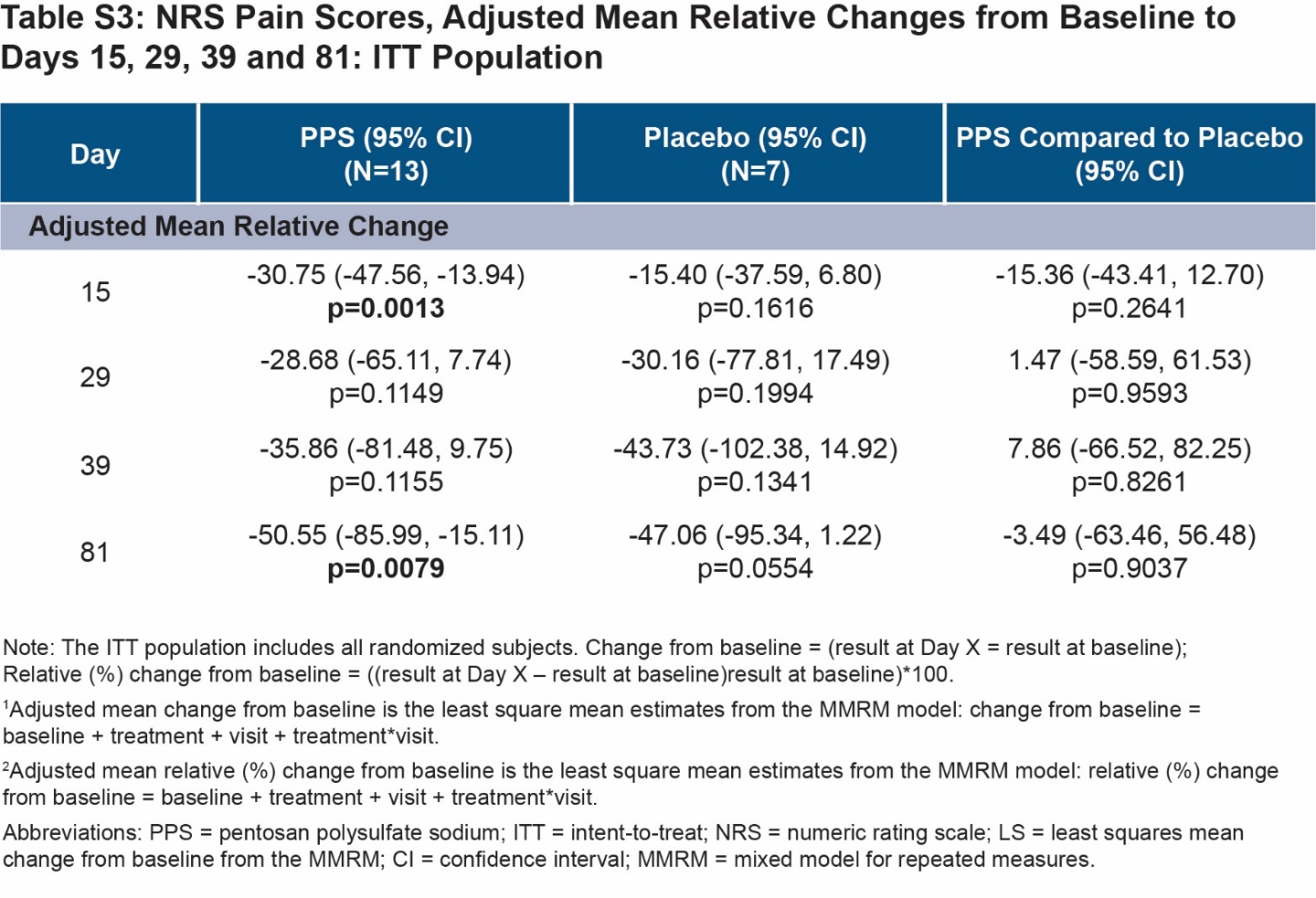


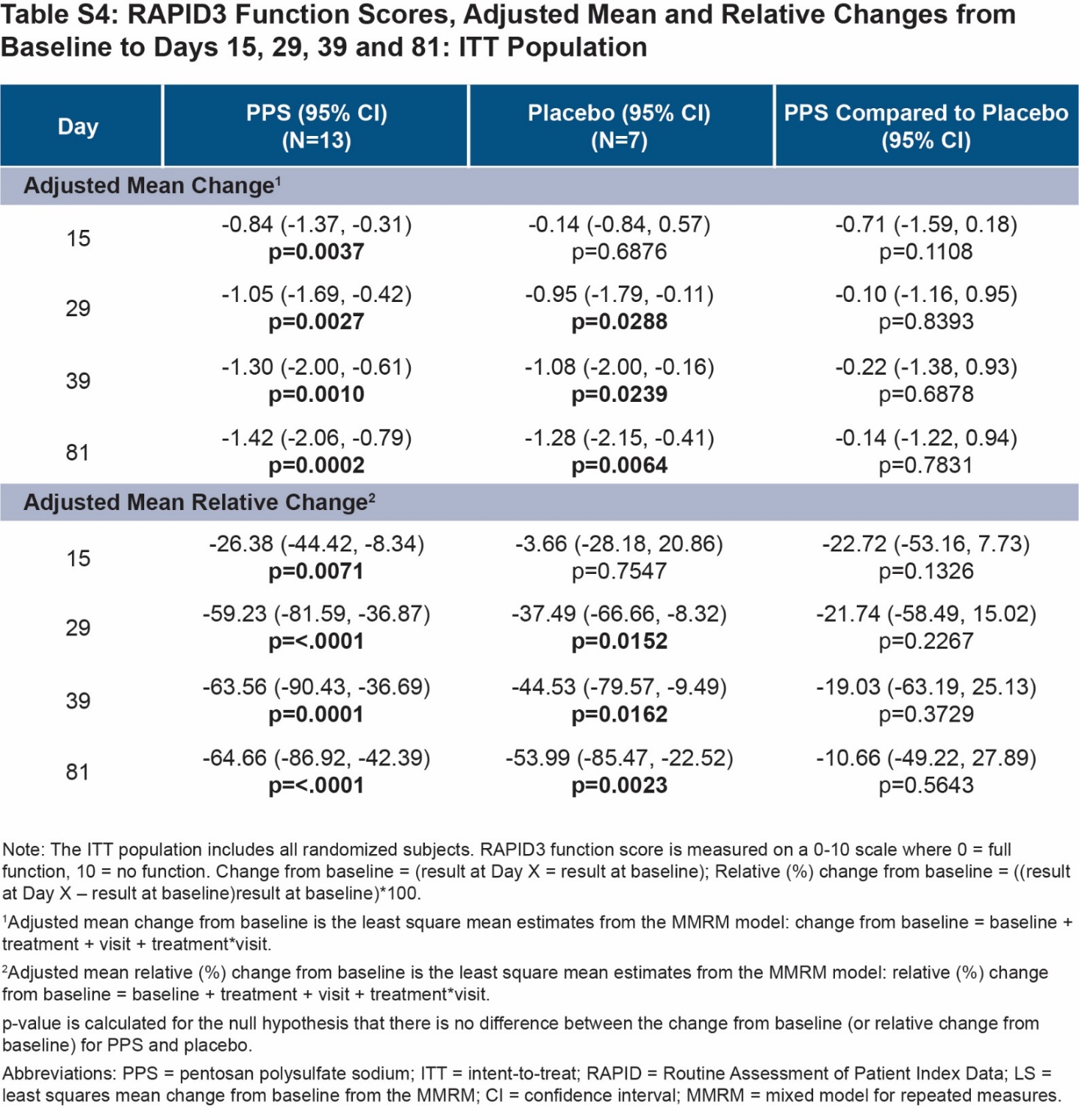


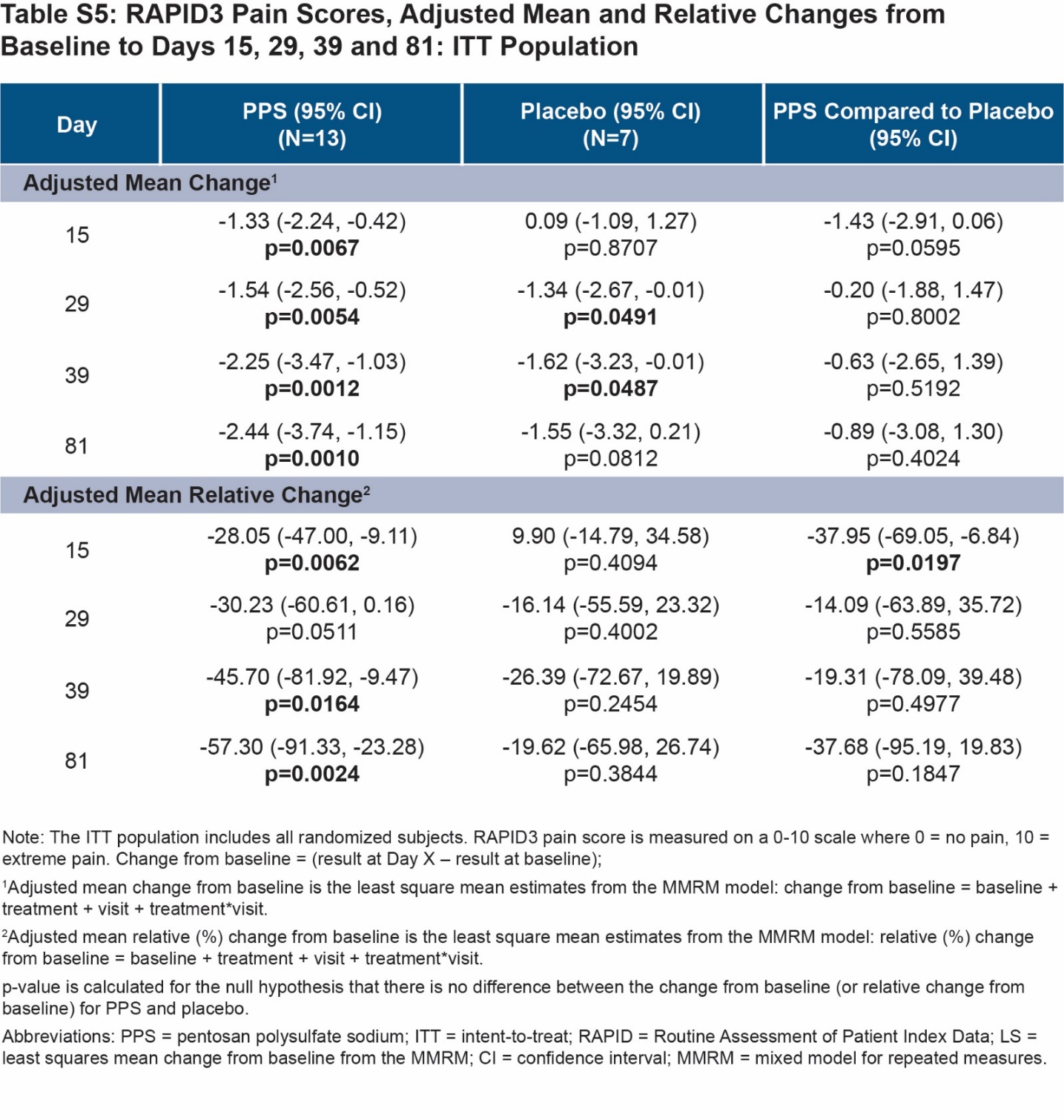


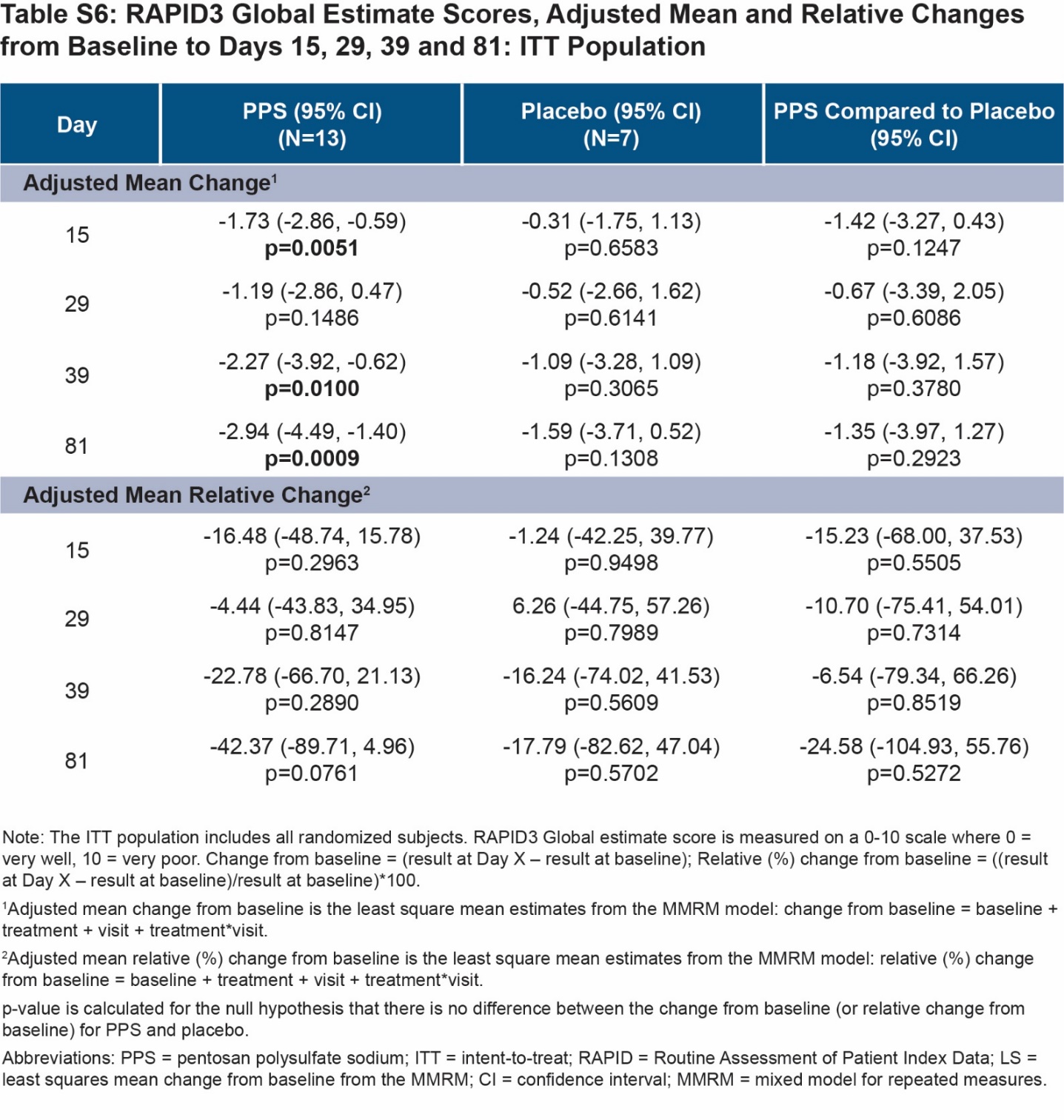


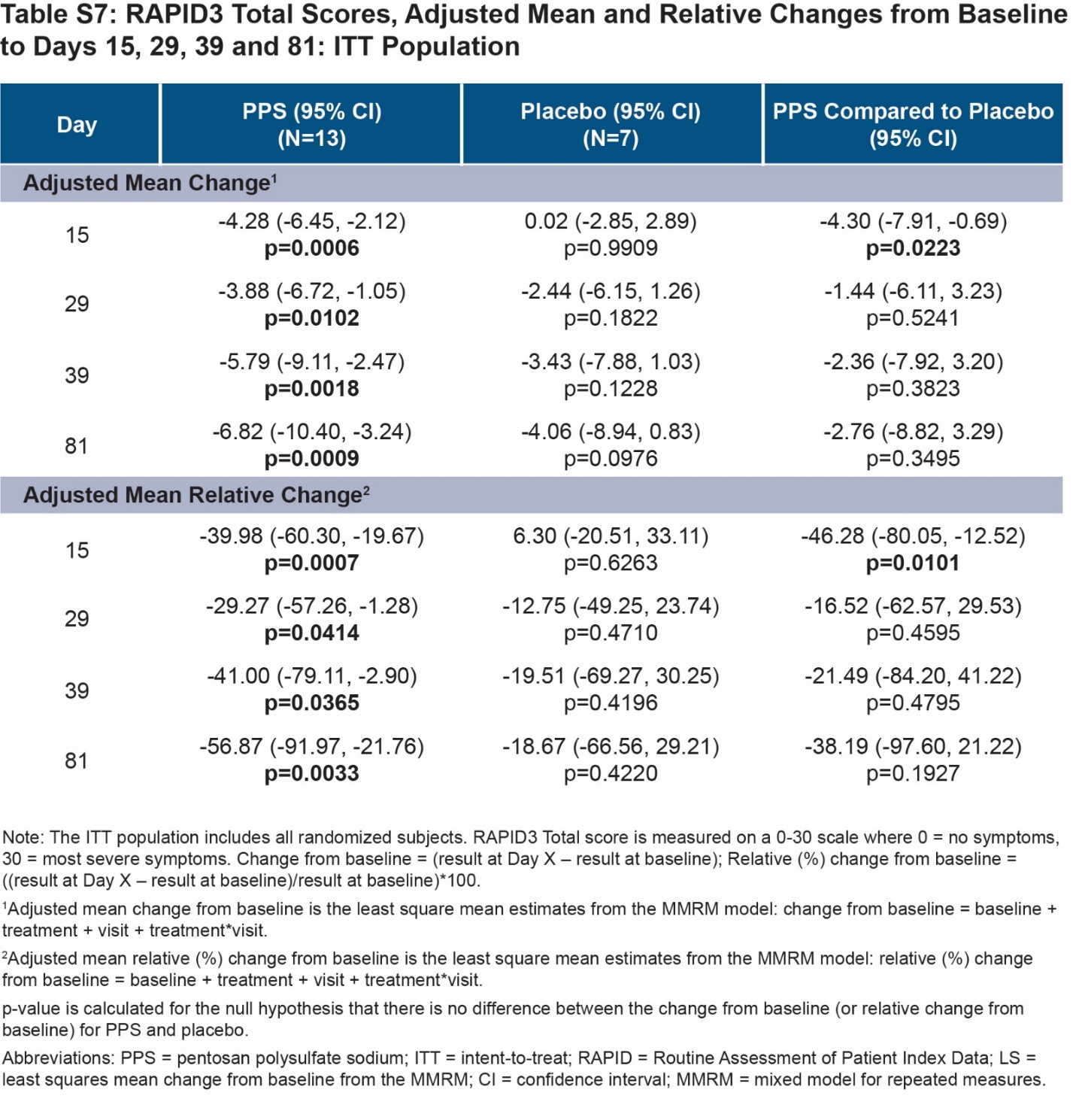


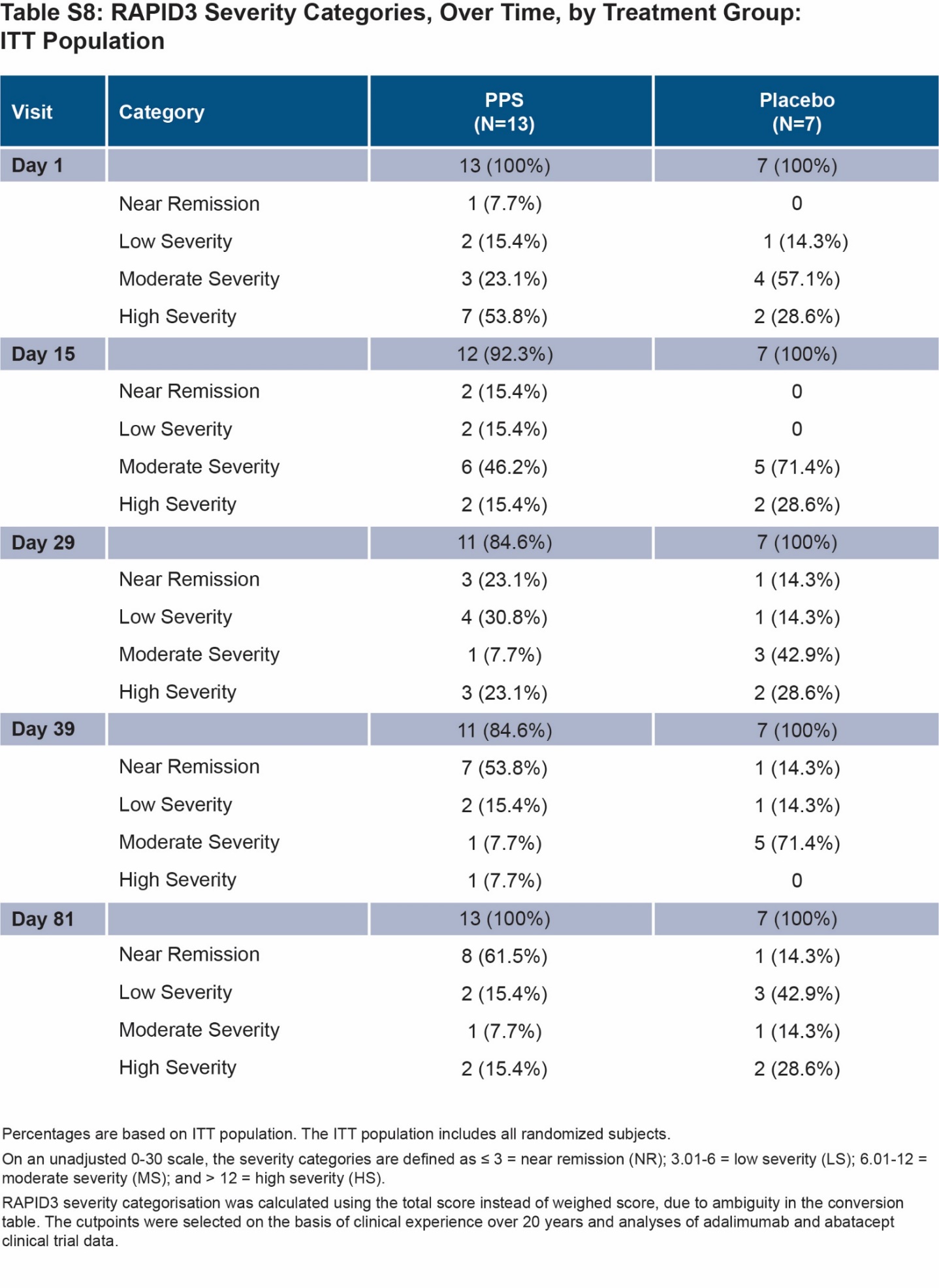


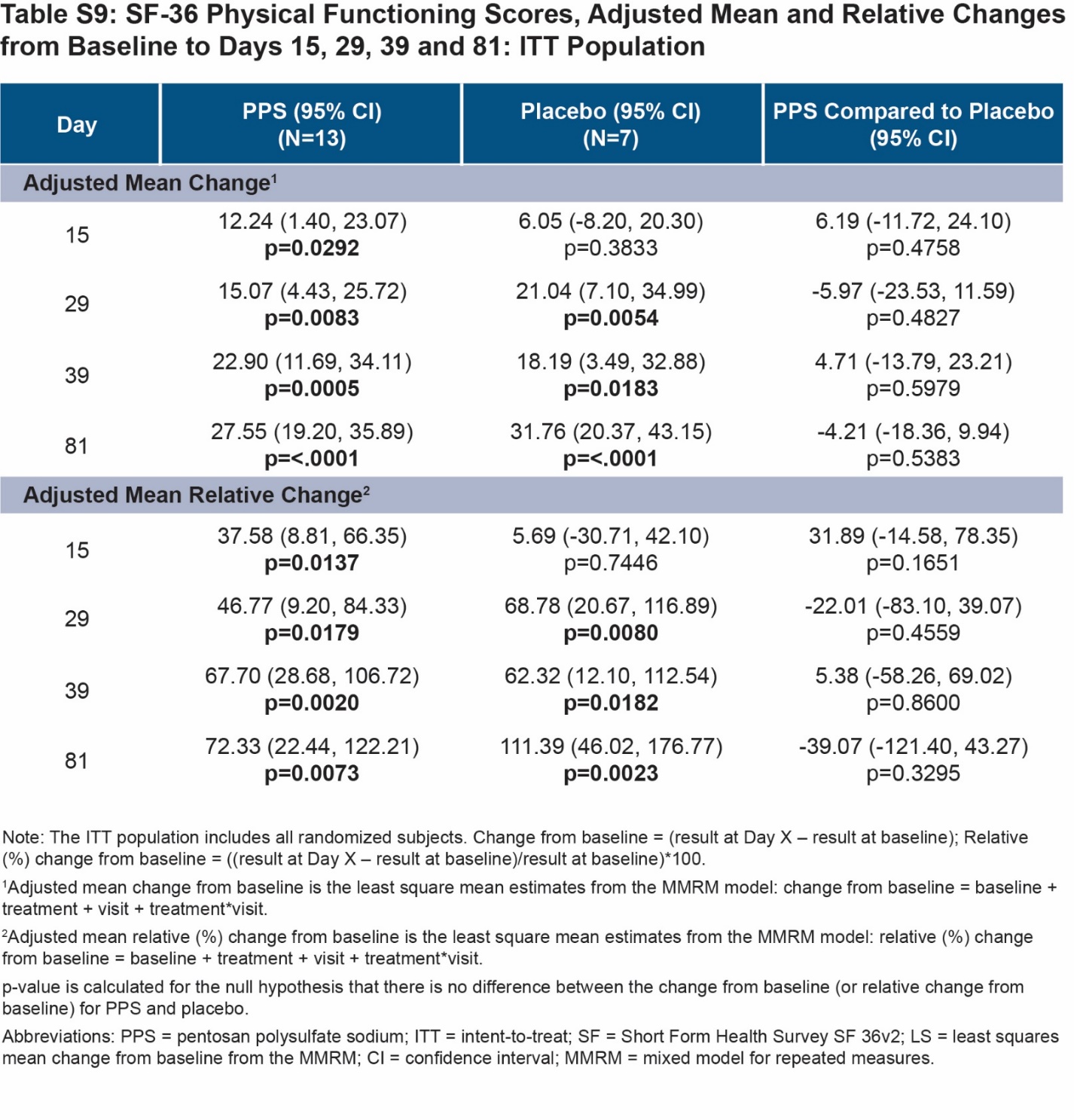


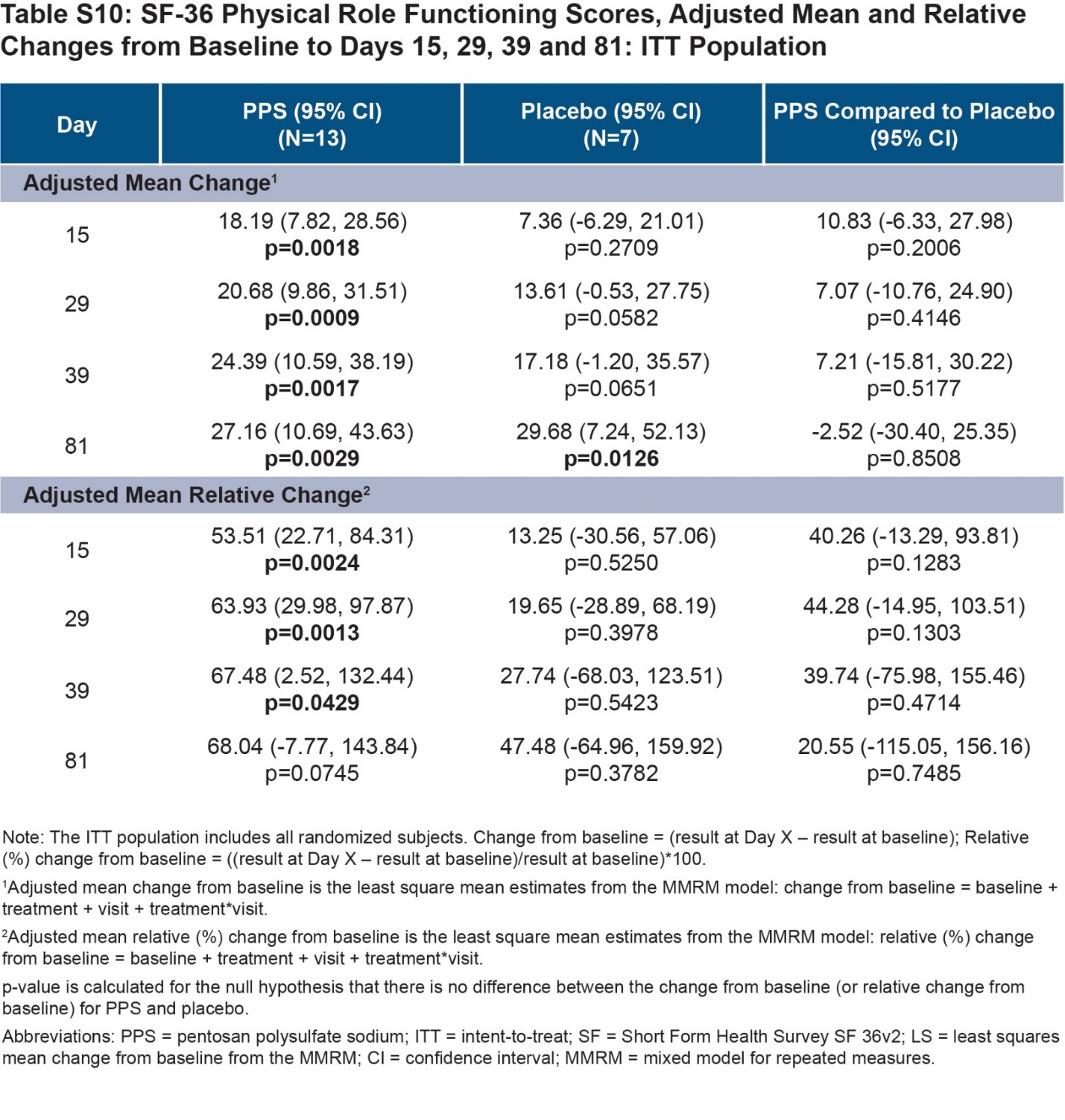


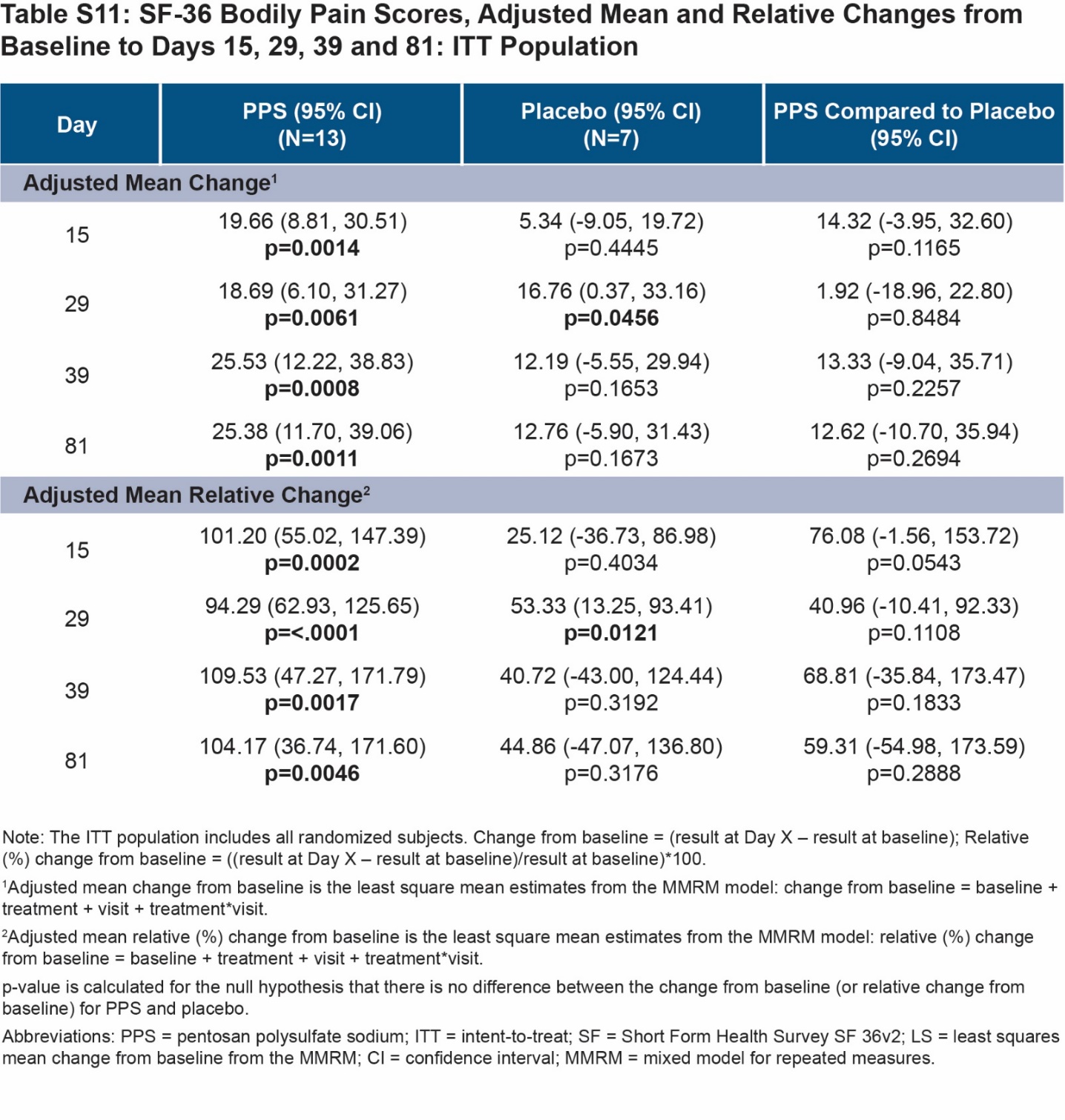


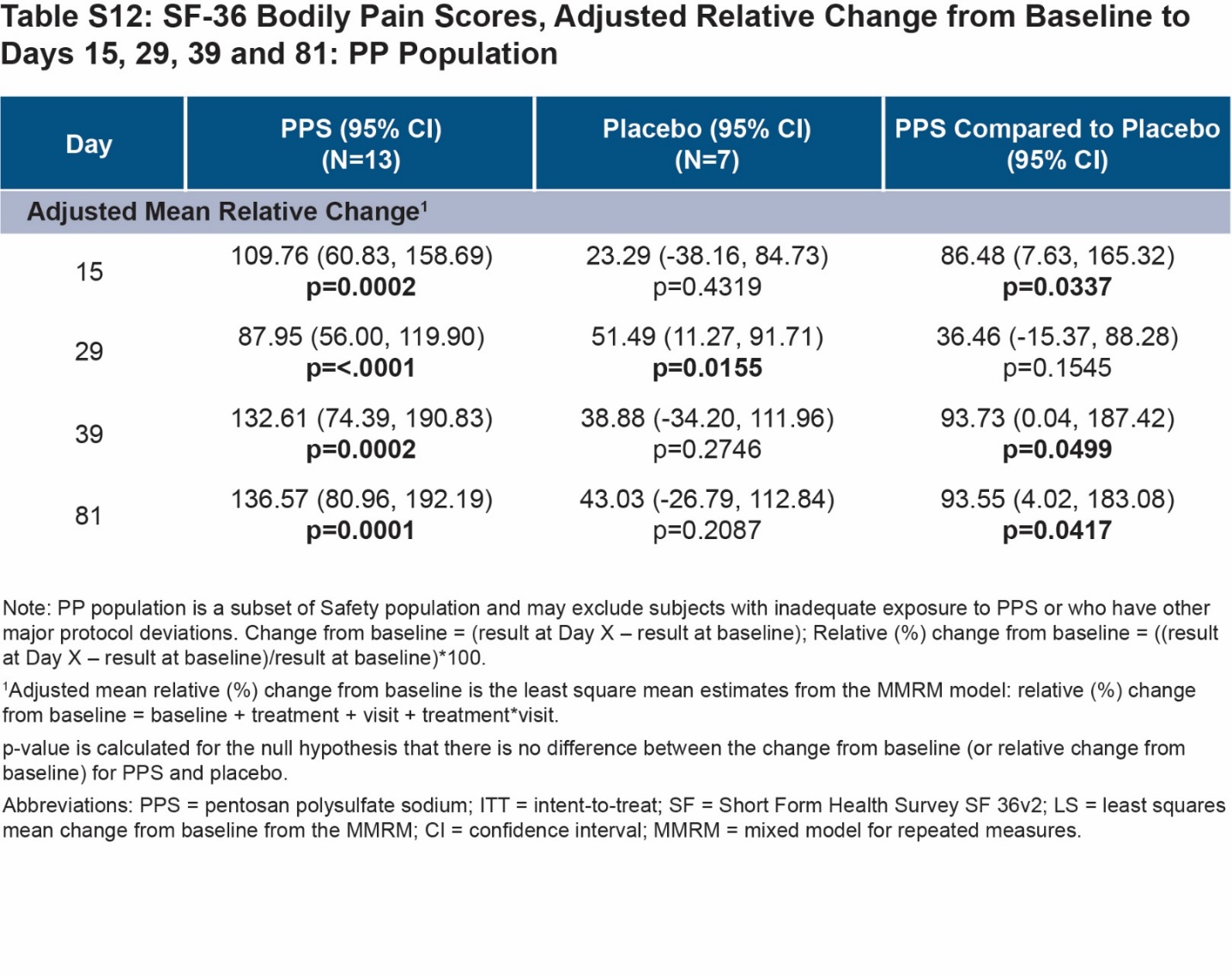


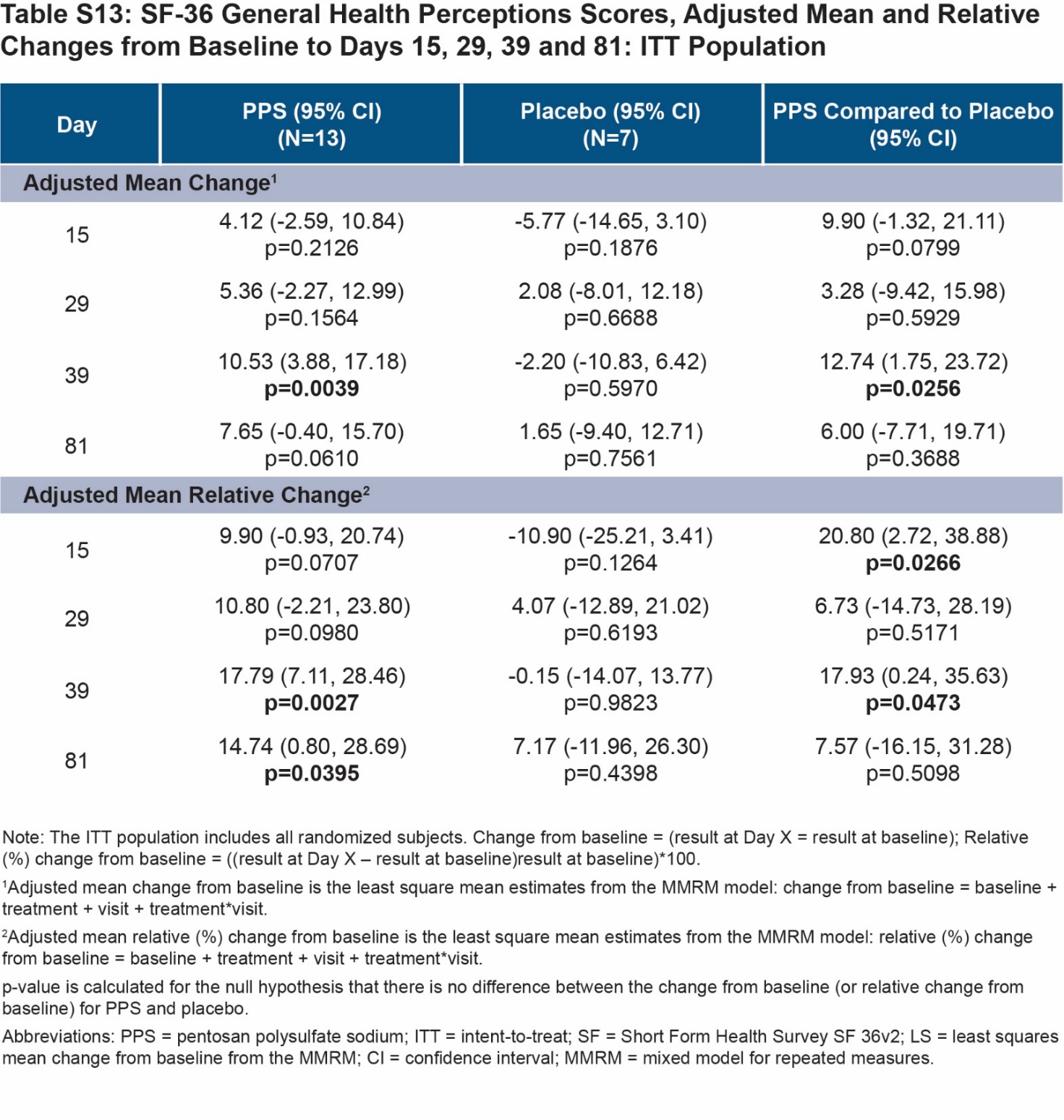


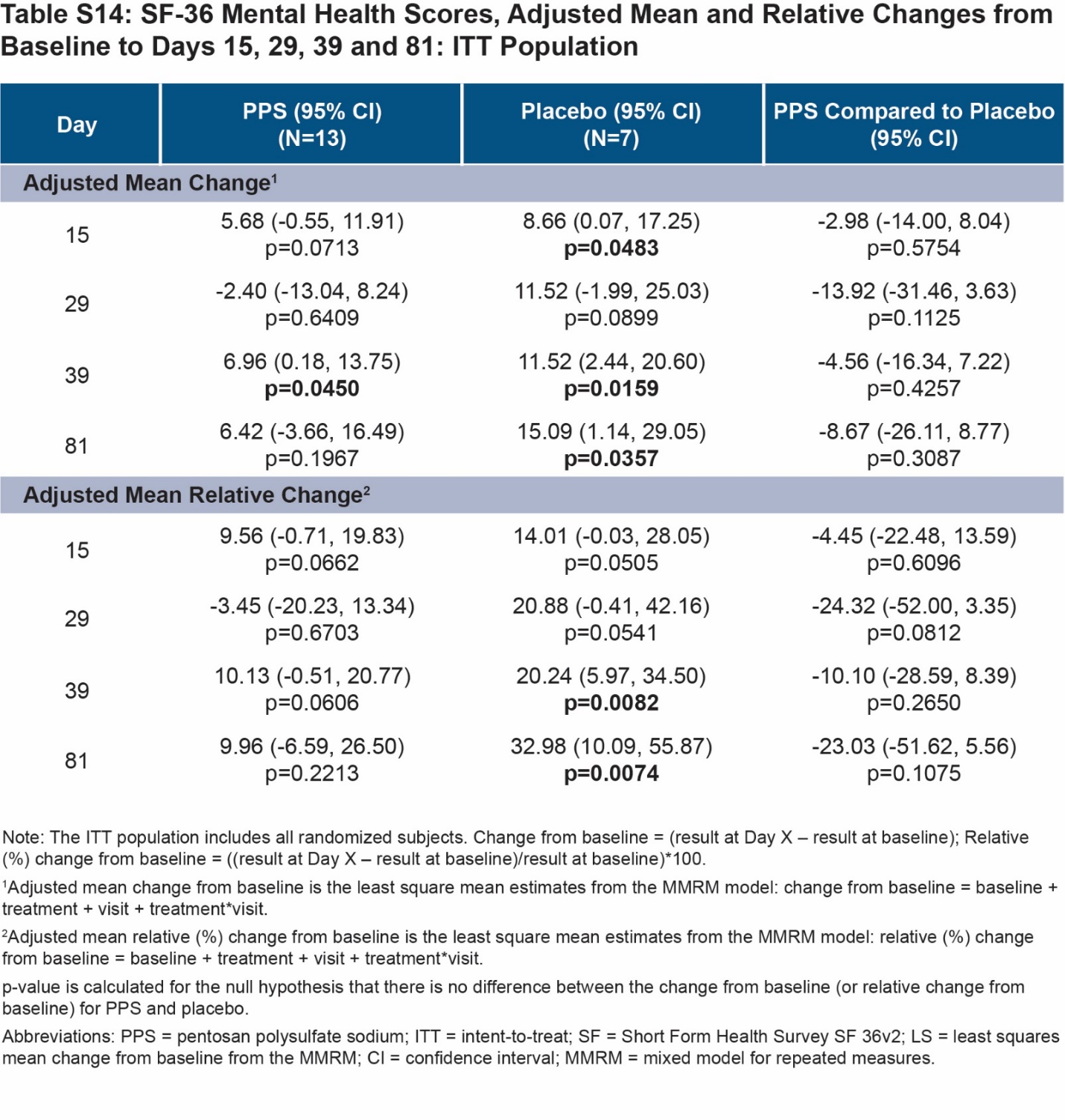


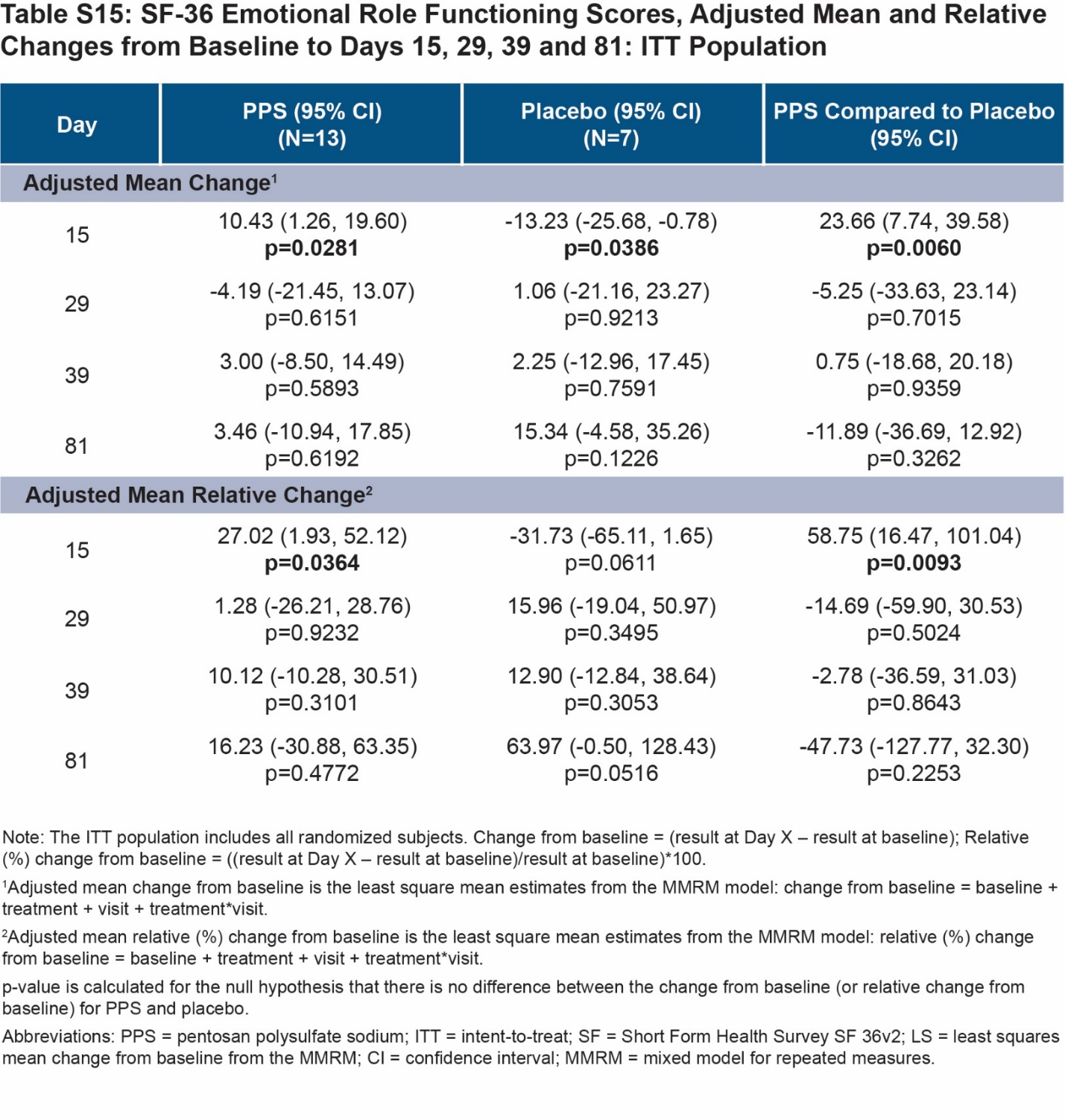


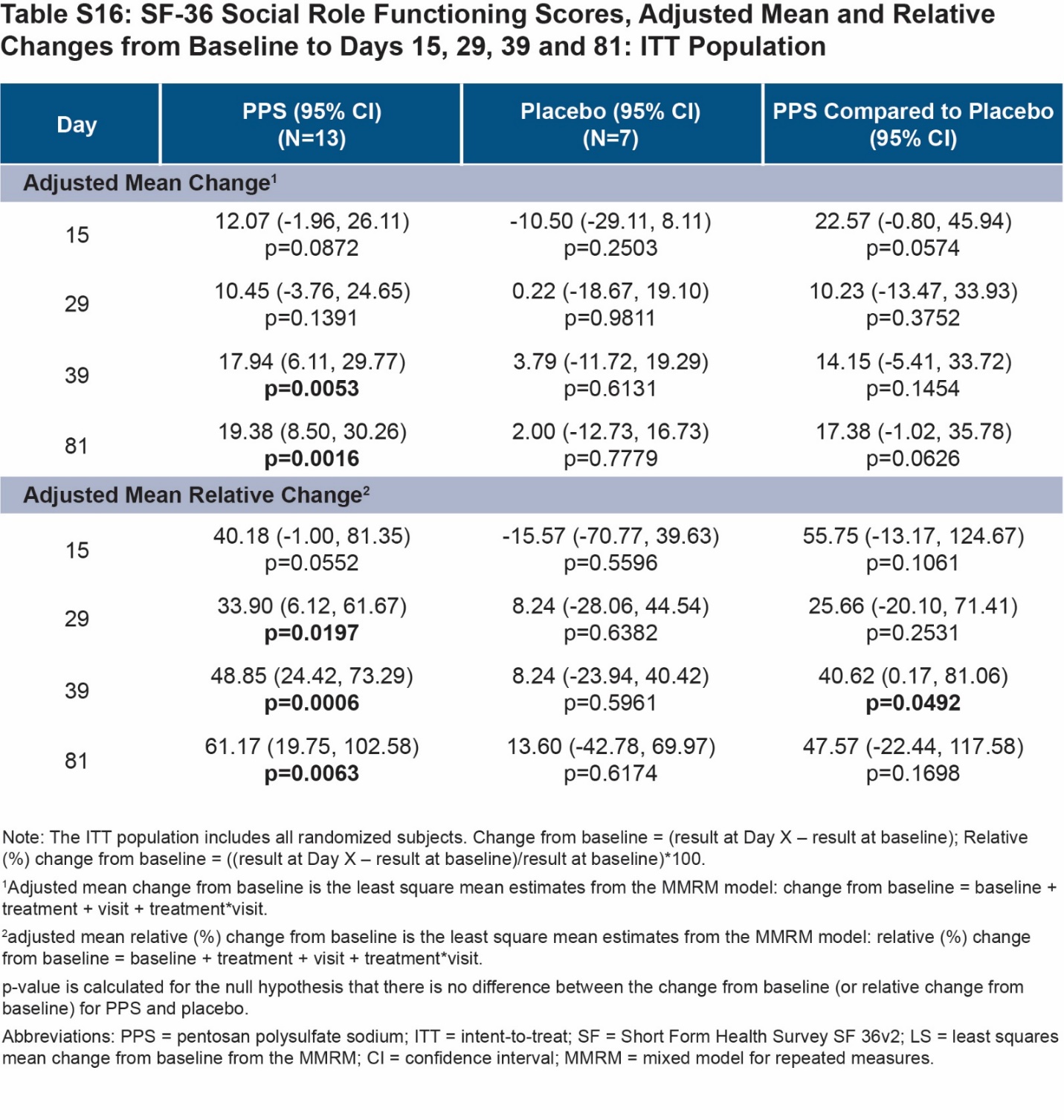


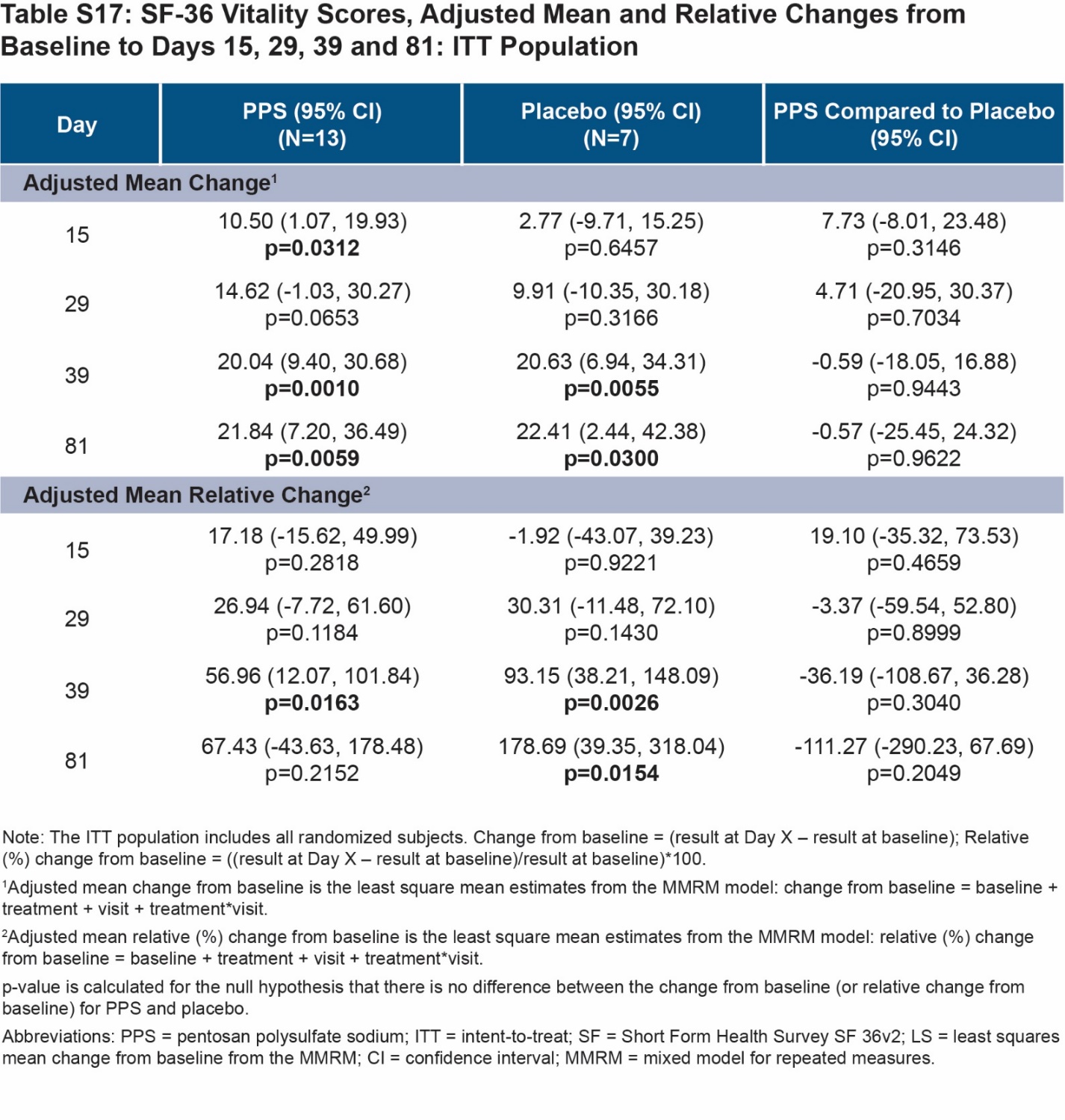


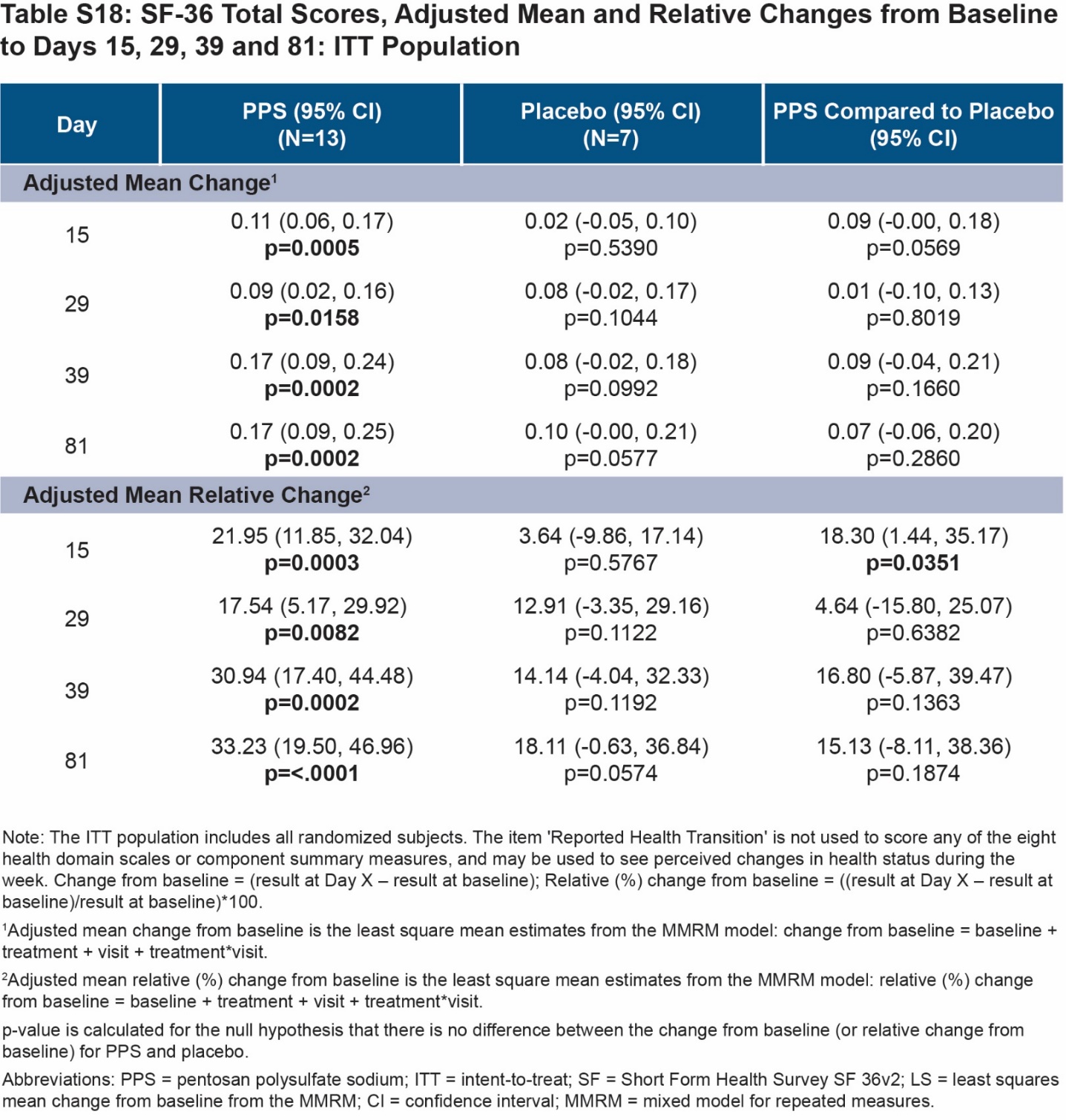


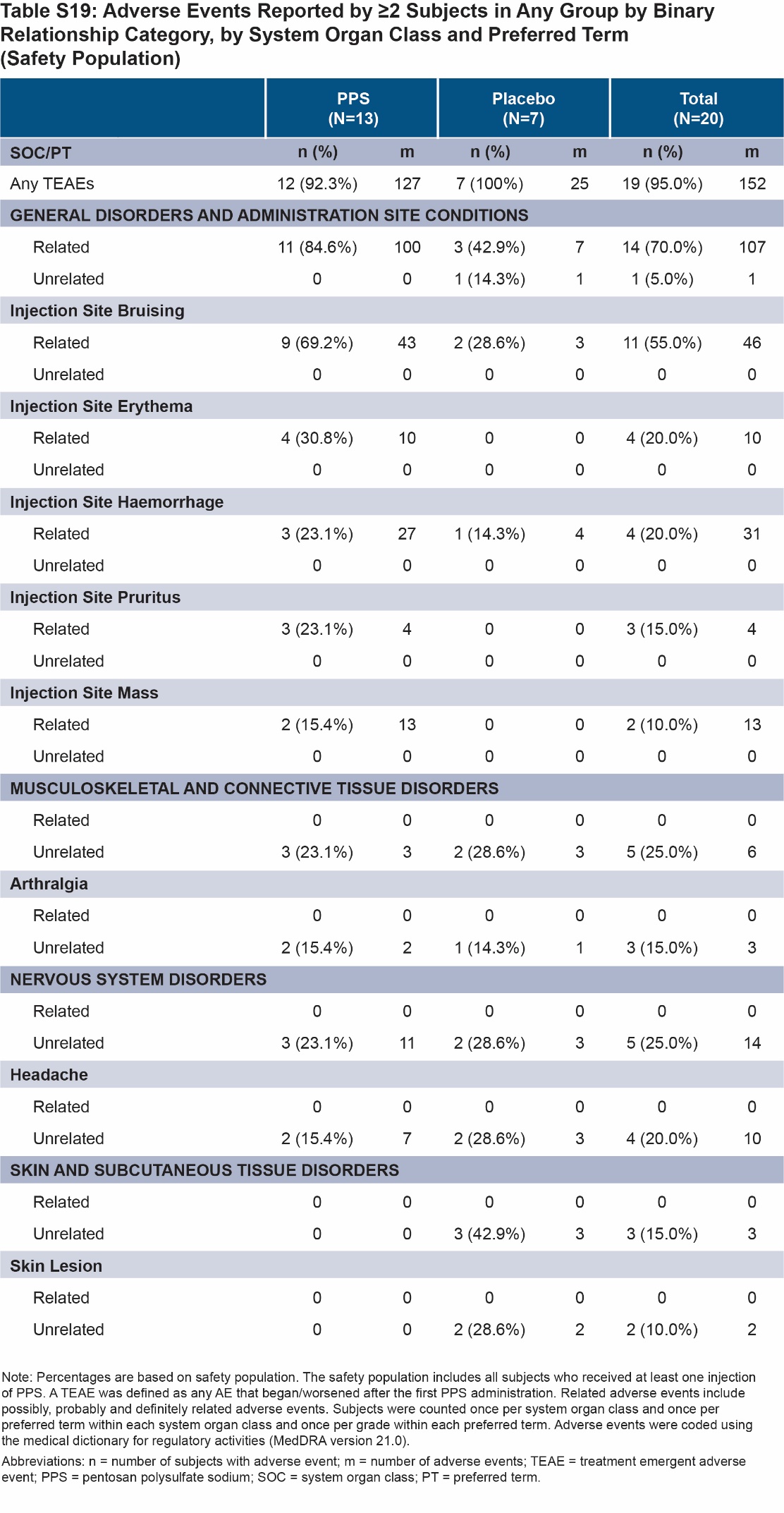


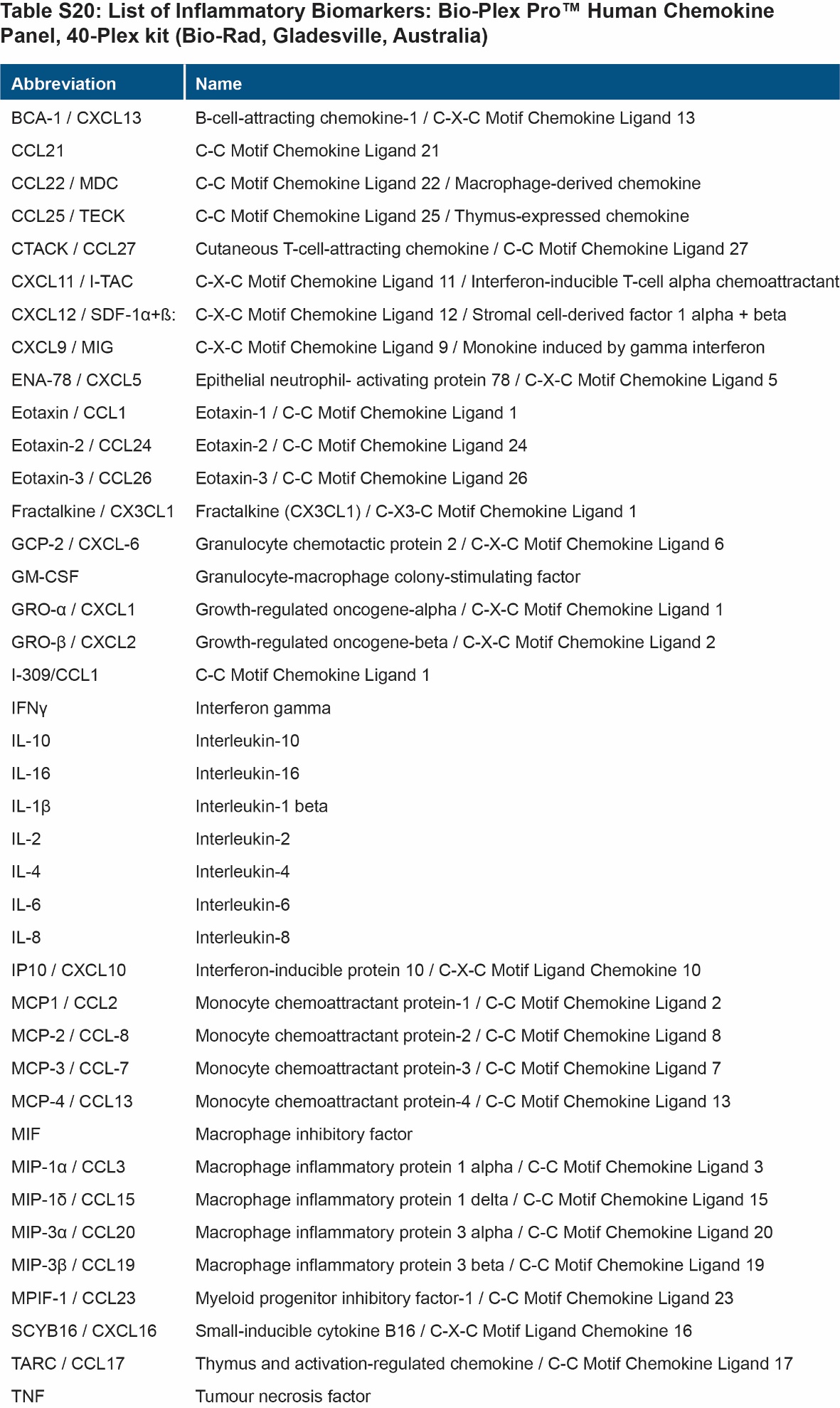

Supplement: Supplementary file 1 — Additional file 1. [file 12891_2021_4123_MOESM1_ESM.docx]
